# *Colletotrichum* Species Associated with Apple Bitter Rot and *Glomerella* Leaf Spot: A Comprehensive Overview

**DOI:** 10.3390/jof10090660

**Published:** 2024-09-19

**Authors:** Vojislav Trkulja, Bojana Čojić, Nenad Trkulja, Andrija Tomić, Slavica Matić, Jela Ikanović, Tatjana Popović Milovanović

**Affiliations:** 1 Agricultural Institute of Republic of Srpska, Knjaza Milosa 17, 78000 Banja Luka, Bosnia and Herzegovina; vtrkulja@blic.net (V.T.); bojanacojic1996@gmail.com (B.Č.); 2 Faculty of Agriculture, University of Banja Luka, Bulevar Vojvode Petra Bojovića 1A, 78000 Banja Luka, Bosnia and Herzegovina; 3 Institute for Plant Protection and Environment, Teodora Drajzera 9, 11040 Belgrade, Serbia; trkulja_nenad@yahoo.com; 4 Faculty of Agriculture, University of East Sarajevo, Vuka Karadžića 30, 71123 East Sarajevo, Bosnia and Herzegovina; tomic_andrija@yahoo.com; 5 Institute for Sustainable Plant Protection, National Research Council, 10135 Turin, Italy; slavica.matic@ipsp.cnr.it; 6 Faculty of Agriculture, University of Belgrade, Nemanjina 6, 11080 Belgrade, Serbia; jela@agrif.bg.ac.rs

**Keywords:** apple, bitter rot, *Glomerella* leaf spot, *Colletotrichum* spp.

## Abstract

Species of the genus *Colletotrichum* are among the most important plant pathogens globally, as they are capable of infecting many hosts—apple (*Malus* spp.) and other fruit and woody plant species—but also vegetable crops, cereals, legumes, and other annual and perennial herbaceous plants. The apple (*Malus* spp.) is attacked by various species from the genus *Colletotrichum*, whereby 27 different species from this genus have been described as the causative agents of apple bitter rot (ABR) and 15 as the cause of *Glomerella* leaf spot (GLS). These species generally belong to one of three species complexes: *Colletotrichum acutatum*, *Colletotrichum gloeosporioides*, and *Colletotrichum boninense*. The largest number of apple pathogens of the genus *Colletotrichum* belong to the species complex *C. acutatum* and *C. gloeosporioides*. However, further data on these species and the interactions between the species complexes of the genus *Colletotrichum* that cause these two apple diseases is needed for the development of effective control measures, thus ensuring successful and profitable apple cultivation. To contribute to this endeavor, a comprehensive review of the causative agents of ABR and GLS from the genus *Colletotrichum* is provided. In addition to presenting the species’ current names, distribution, economic significance, and the symptoms they cause in apple, their development cycle, epidemiology, and molecular detection strategies are described, with a particular emphasis on control measures.

## 1. Introduction

Although *Colletotrichum* species are capable of infecting many hosts, their impact on apple [*Malus domestica* (Suckow) Borkh.] is of particular economic significance globally due to the significant yield losses caused in this species [1]. An ample body of evidence indicates that different species of the genus *Colletotrichum* can cause disease symptoms on the same host [1,2,3], and can also cause bitter rot in fruit and glomerella leaf spot (GLS) [4,5,6,7]. As these pathogens are capable of infecting over 100 fruit and vegetable species, it is not uncommon for one species to be present on multiple hosts or for multiple species to coexist on the same host [1,3,8,9,10].

Nonetheless, the interactions between species complexes of the genus *Colletotrichum* that cause apple bitter rot (ABR) and GLS are insufficiently explored [3]. This is a significant shortcoming, as understanding each species’ response to different control measures will contribute to more effective and profitable apple cultivation. For instance, the species *C. gloeosporioides* is highly sensitive to certain fungicides from the benzimidazole group, while *C. acutatum* exhibits only a moderate sensitivity [11]. Elucidating these differences, along with the accurate identification of species within this genus and their occurrence in different hosts, is crucial for the appropriate selection of fungicides or apple varieties with sufficient resistance [12].

To aid in this effort, the species of the genus *Colletotrichum* that are known causative agents of ABR and GLS in apple worldwide are described, including their current names, distribution, economic significance, and the symptoms they cause in apple, along with their development cycle, epidemiology, molecular detection, and effective measures for their control.

## 2. History of Occurrence Apple Bitter Rot and *Glomerella* Leaf Spot

ABR and its causative agent were first described by Berkley in 1856 based on observations in England [13]. However, referring to an unpublished manuscript dating from 1829, Walker [14] argued that the same disease, under the name “anthracnose”, was described in Europe much earlier.

Although the disease was also identified in the United States in 1867, its first description pertaining specifically to this country dates back to 1874 [15,16]. Still, Anderson [17] is of the view that, due to its extreme harmfulness, this disease was probably well known in the United States much earlier. To support this claim, this author refers to an unpublished manuscript written by Coxe before 1828, offering an accurate description of this disease and its impacts on apple orchards near Philadelphia, Pennsylvania. Anderson [17] further notes that, in 1850, Baker already recognized that an apple variety commonly grown in Morgan County, Ohio, was susceptible to anthracnose, suggesting that bitter rot was likely present on the American continent in early 19th century. These suppositions are supported by other authors [18,19,20,21,22].

Although in this early period bitter rot in apple was commonly known as “anthracnose”, it cannot be established with certainty whether this term was used exclusively for apple or more generically. Similarly, there is no consensus on the first occurrence of “bitter rot”, but it is believed that this term was coined to describe the bitterness of the diseased fruits [23]. On the other hand, Galloway [24] and Southworth [25] opted for “ripe rot” instead, due to the assumption that only ripe fruits are susceptible to this disease, given that the same observation has been made for grapes. The fact that these names were used synonymously prior to the 20th century is also confirmed by Alwood [23].

GLS on the Gala and Golden Delicious apple cultivars was first identified and described by Leite et al. [26] in the state of Paraná, Brazil, who determined that this disease is caused by a fungus associated with the sexual stage of the fungus *Colletotrichum gloeosporioides*. This is also the first report indicating that fungi from the *Colletotrichum* genus can lead not only to ABR but also cause apple leaf disease.

## 3. History of Taxonomy *Colletotrichum* Species Causing Apple Bitter Rot and *Glomerella* Leaf Spot

Further advances in this domain were made by M.J. Berkeley, who described several fungi causing fruit rot on various fruit species. In 1854, this author described the species *Septoria rufo-maculans* Berk. as a causative agent of rot in ripe grapes [27], which he renamed *Ascochyta rufomaculans* (Berk.) Berk. in 1860 [16,23,28,29]. Von Thümen [30] later classified the fungus *Ascochyta rufomaculans* into the genus *Gloeosporium*, and its name was subsequently changed to *Gloeosporium rufomaculans* (Berk.) Thüm.

Berkeley [31] was the first to describe the causal agent of ABR, identifying it as *Gloeosporium fructigenum* Berk. In 1859, Berkeley also identified the species *Gloeosporium laeticolor* Berk. as a new fungus causing rot in peach and nectarine fruits [32]. *Gloeosporium versicolor* was also used by Curtis [33] in the catalog of plants of North Carolina to describe a new fungus species present on rotten apple fruits. Nonetheless, Berkeley and Curtis [15] are credited for the first official description of this fungus, due to which it was denoted as *Gloeosporium versicolor* Berk. & M.A. Curtis [16,23].

Experiments that were subsequently conducted by von Thümen [30] indicated that the same fungus causes rot in grape berries and apple fruits. This finding was confirmed by Southworth in 1891 [25] through inoculation experiments, as a part of which grape berries were inoculated with spores of the fungus *Gloeosporium fructigenum* (the causative agent of ABR), and apple fruits were inoculated with spores of *G. rufomaculans* (the causative agent of ripe rot in grape berries). As the inoculation led to the emergence of rot in both cases, this was sufficient evidence to confirm that it was caused by the same fungal species [25]. Furthermore, Halsted’s experimental findings [34] confirm Southworth’s results demonstrating that the same fungus also causes ripe rot in quince, pear, peach, nectarine, pepper, and other plants. Further studies revealed that *Gloeosporium fructigenum*, *G. rufomaculans*, *G. versicolor*, and *G. laeticolor* are the same fungal species [16]. Since these four fungi are the same species according to the International Code of Nomenclature for algae, fungi, and plants (ICNafp), *Gloeosporium rufomaculans* (Berk.) Thüm. (but not *G. fructigenum*) was taken as the valid name for the asexual phase of the causative agent of ABR, until the revision of species names within the genus *Gloeosporium* by von Arx [35,36].

The sexual (teleomorphic) stage of the fungus *Gloeosporium fructigenum*, the causative agent of bitter rot, was first discovered and described on apple fruit by Clinton [37] in Illinois, US. This author placed the new fungus in the genus *Gnomoniopsis*, described by Stoneman [38] after obtaining the sexual stage of the fungus in cultures. Stoneman subsequently included into the genus *Gnomoniopsis* the sexual stages of four fungus species that were previously known only in asexual stages: *Gloeosporium cingulata* G.F. Atk., *Gloeosporium piperatum* Ellis & Everh., *Colletotrichum cinctum* (Berk. & M.A. Curtis) Stoneman, and *Colletotrichum rubicola* Ellis & Everh. Clinton included in this group the fungus *Gloeosporium fructigenum*, the causative agent of ABR, and therefore named the sexual stage of this fungus *Gnomoniopsis fructigena* Clinton.

Earlier, Atkinson [39] described a fungus parasitizing privet (*Ligustrum vulgare* L.), *Gloeosporium cingulatum* G.F. Atk., as the causal agent of the anthracnose. When Stoneman [40] later obtained the sexual stage of this fungus, he described it as *Gnomoniopsis cingulata* (G.F. Atk.), but also stated that it is identical to the species causing ABR.

However, when establishing the genus *Gnomoniopsis*, Stoneman [40] overlooked the fact that five years before her publication, Berlese [41] used the name *Gnomoniopsis* for a genus of fungi very different from the sexual stage of the genus *Gloeosporium* that she described, which is why Schrenk and Spaulding [42] proposed a new name, *Glomerella,* for the genus *Gnomoniopsis*.

Therefore, according to the ICNafp rules, since the use of *Gnomoniopsis cingulata* preceded Clinton’s proposal, in 1903, *Glomerella cingulata* (G.F. Atk.) Spauld. & H. Schrenk became the officially valid name for the species causing ABR [42].

Nonetheless, *Gloeosporium fructigenum*, as the causal agent of ABR, remained in prevalent usage well into the 1980s [14,17,43,44,45], despite the fact that, during the revision of the genus *Gloeosporium*, von Arx [35,36] determined that *G. fructigenum* is one of several synonyms for *Colletotrichum gloeosporioides* (Penz.) Penz. & Sacc., the anamorph of the species *Glomerella cingulata*.

Accordingly, for a more comprehensive understanding of the causative agents of ABR, it is essential to make a distinction between the genera *Gloeosporium* and *Colletotrichum*, as reflected in the current taxonomy of the genus *Gloeosporium* Desm. & Mont., which has undergone significant changes since its first description in 1849 by Desmazieres and Montagne [46].

The most complete explanation of the need for revision, as well as the actual revision of species from the genus *Gloeosporium*, was given by von Arx in 1957 [35,36], who was also responsible for a new classification, as a part of which many species from the genus *Gloeosporium* were transferred to the genus *Colletotrichum* [36].

Namely, for the genus *Gloeosporium*—which is considered very close to the genus *Colletotrichum*—there was a long-held assumption that certain types of phytopathogenic fungi belong to the genus *Gloeosporium* or the genus *Colletotrichum* based on the presence or absence of setae. According to this criterion, phytopathogenic fungi without conidiomatic setae are classified in the genus *Gloeosporium*, while those with setae belong to the genus *Colletotrichum*. However, such a separation of the genus *Gloeosporium* from the genus *Colletotrichum* is based on a wrong premise, because several authors have established that the formation or non-formation of setae on acervulae is an unreliable taxonomic criterion, since their formation is often variable and depends on environmental factors [47,48,49], most likely atmospheric moisture. Therefore, it can be expected that, under different growing conditions, some species will form setae in culture, and others will not. This is the main reason why numerous species described as *Gloeosporium* spp. actually belong to the genus *Colletotrichum* [36,50]. As for the absence of setae on the conidiomata in some *Colletotrichum* species, it is considered that this trait is to some extent genetically inherited, because some species, such as *C. musae* and *C. gossypii* var. *cephalosporioides* A.S. Costa, never form setae [51,52].

Representatives of the genus *Colletotrichum* are facultative pathogens capable of causing a variety of symptoms on their hosts. However, it was previously common practice to name species within this and similar genera based on their host plants [35]. Due to this erroneous approach, many species were known under different names, resulting in about 750 species that were later reduced to only 11 (along with 11 host-specific forms) once the morphological species concept was adopted [36]. In these more comprehensive analyses, focus was given to morphological differences such as conidial size and shape, the presence or absence of setae, the presence or absence of sclerotia, colony color and growth rate, and the existence of teleomorphs.

Prior to their adoption, the species *C. gloeosporioides* used to be known under around 600 synonyms, most of which were proposed by von Arx [36,50]. This causative agent of ABR was separated within the genus *Colletotrichum* to denote the extremely variable conidial stage of the teleomorph *Glomerella cingulata*. However, this nomenclature was later revised by Sutton [52,53,54] based on the premise that many of the recommended synonyms were grounded in insufficient experimental evidence, inadequate observations, or the incorrect interpretation of original material. Subsequent detailed studies on the morphological, cultural, and pathogenic characteristics of individual representatives led to the addition of several new species into the genus *Colletotrichum*. For example, von Arx [55] increased the number of species within this genus to 25, while Sutton [54] initially listed 22 and later increased this number to 39 [52]. However, it soon became evident that morphological and cultural characteristics were insufficient for the identification and classification of species, given that in many cases—including the *Colletotrichum* species complex—morphological characters overlap [56]. This recognition prompted extensive research on this fungal genus, resulting in several taxonomic revisions. Still, Hyde et al. [56], who provided a comprehensive overview of this complex, including 66 accepted and 20 dubious species names, was the first to call for the use of molecular methods for species classification. With the advent of multilocus phylogenetic analysis, a large number of species within the genus *Colletotrichum* could finally be described. Using this strategy, Cannon et al. [2] listed 119 accepted species within the genus *Colletotrichum*, classified into nine species complexes, while [57] listed 190 accepted species in this genus, classified into 11 species complexes. Only five years later, Jayawardena et al. [58] listed 248 accepted species within this genus, 235 of which belong to 14 species complexes, while the remaining 13 remain unclassified. Available evidence also indicates that ABR and GLS in apple are primarily caused by species of the genus *Colletotrichum* belonging to either *C. acutatum* or *C. gloeosporioides*, but also the *C. boninense* species complex.

The adoption of the Melbourne Code by the International Botanical Congress held in Melbourne in 2011 also exerted significant influence on the fungal naming practice. Not only was the International Code of Botanical Nomenclature (ICBN) renamed as the International Code of Nomenclature for algae, fungi, and plants (ICNafp), but the “one fungus—one name” principle was also adopted [59,60,61,62]. In accordance with Article 59 of this Code, the International Subcommission on the Taxonomy of *Colletotrichum* (ISTC) was established. At its inaugural meeting held on 9 August 2012, in Beijing, China, all ISTC members supported the preferential use of the asexual name of the genus *Colletotrichum* (1831) relative to *Glomerella* (1903), justifying this decision by the prevalent use of *Colletotrichum* in applied sciences [63,64].

GLS is a relatively new disease in apple compared to bitter rot. Caused by a strain of *Glomerella cingulata*, this disease was first reported in the United States by Taylor [65], who noted that the necrotic spots on apple leaves and ABR in fruit were caused by a *G. cingulata* strain that differed from the common isolates of the fungus causing bitter rot. The author cited pronounced differences with respect to its ability to overwinter on apple leaves, cause leaf spots, and produce characteristic symptoms on fruit without sporulation in fruit lesions [65]. However, Taylor named the disease caused by *G. cingulata* as necrotic leaf spots, even though a similar name was already used for a physiological disorder in Golden Delicious apples. Although this oversight led to confusion among apple growers and researchers, this naming issue did not become a serious concern until leaf spot disease became widespread on Gala apple cultivars in Brazil, where it was denoted as “*mancha foliar de Glomerella*” (*Glomerella* leaf spot) [26]. To resolve this issue, Sutton and Sanhueza [66] proposed using more precise terminology to differentiate diseases that produce similar symptoms but have different causes. Accordingly, in Brazil, necrotic leaf spot of Golden Delicious was adopted for the physiological disorder, while *Glomerella* leaf spot (GLS) was chosen for the disease caused by the fungus *G. cingulata*, which was also adopted in the US [66,67].

Although the temporal origin of GLS remains relatively underexplored, the three hypotheses regarding its emergence in the US proposed by González et al. [68] are noteworthy. According to the first hypothesis, the disease originated from an endemic population infecting fruit, most likely from a population of *G. cingulata* that was initially only pathogenic to apple. This hypothesis is supported by similarities in mtDNA haplotypes between isolates infecting fruit and those infecting leaves. Adherents to this perspective have also suggested that the Gala cultivar could have acted as a selective factor, favoring genotypes capable of causing GLS. The second hypothesis suggests that isolates infecting fruit originated from a leaf-infecting population. However, this view has limited empirical support given that bitter rot has been present for many years in the US, whereas GLS is a new disease [67]. According to the third hypothesis, genotypes causing GLS could have been recently introduced into the population. This perspective is also problematic, as isolates causing GLS in the United States and Brazil have been confirmed to belong to different groups [68].

It is evident that molecular techniques are necessary to precisely identify individual species causing GLS. Progress has already been made in the understanding of the *Colletotrichum* taxonomy, especially the new GLS causative agents. According to the experiments conducted by González [69] and González et al. [68], for example, only specific taxa of *G. cingulata* are responsible for leaf spot on apple, given that other taxa such as *C. gloeosporioides* and *C. acutatum* did not exhibit pathogenicity towards the leaves of tested apple cultivars in the US. However, studies based on advanced techniques—such as multilocus phylogeny, morphological characterization, and pathogenicity tests—have led to the reclassification of several species, including *C. gloeosporioides* and *C. acutatum* [3,9,10]. These approaches have also enabled the identification of new species within these complexes as GLS-causative agents [7,70,71,72,73,74,75,76].

## 4. Symptoms of Apple Bitter Rot

**Figure 1:**
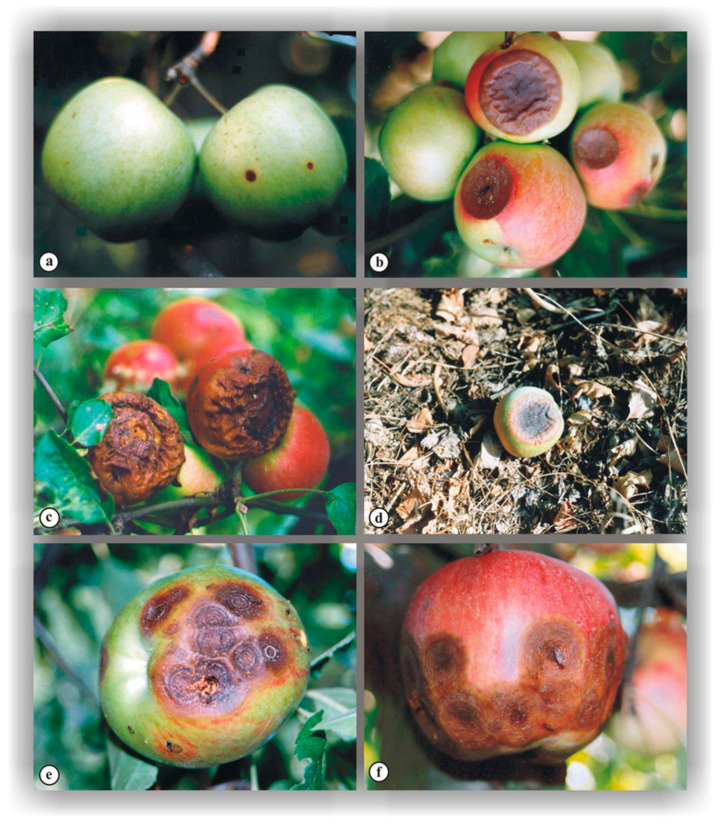
*Colletotrichum* spp. Symptoms of bitter rot on apple fruits in the orchard: (**a**) initial disease symptoms, manifesting as small necrotic spots on the fruit surface; (**b**) further development of the disease showing characteristic symptoms of bitter rot [77]; (**c**) late stage of disease development (bitter rot affects nearly the entire fruit) [77]; (**d**) fallen diseased fruit with evidence of abundant parasite fruiting, serving as a source of inoculum [77]; (**e**) ants and (**f**) flies as vectors of conidia from diseased apple fruits with abundant parasite fruiting [78] (photo V. Trkulja).

Bitter rot initially manifests in apple fruit as small, round spots of light brown color (Figure 1a). Under favorable conditions for pathogen development, these spots grow rapidly, typically becoming completely circular and slightly sunken at the center, forming an easily recognizable “saucer-like” depression on the fruit surface (Figure 1b).

**Figure 2:**
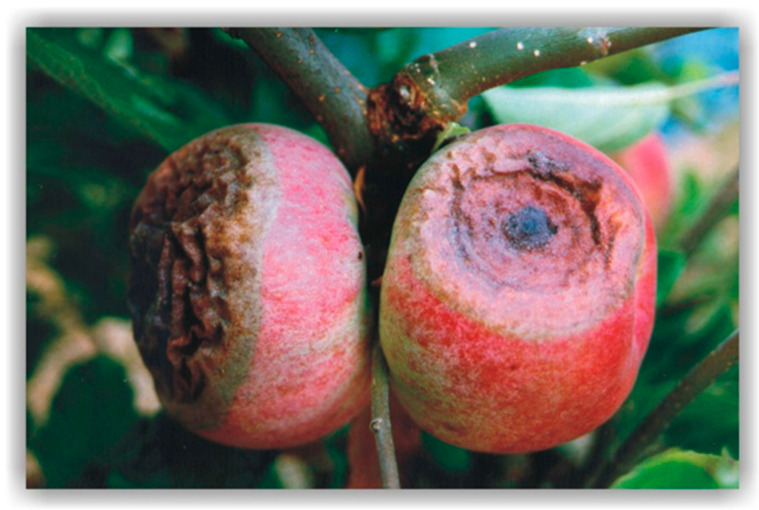
*Colletotrichum* spp. appearance of diseased Red Delicious apple fruits in the orchard exhibiting characteristic bitter rot symptoms [77] (photo V. Trkulja).

Although several spots can develop in a single fruit (Figure 1e,f and Figure 2), in such cases, only a few continue to intensively expand. Spots with a diameter below 1 cm have a smooth surface but typically change color to dark brown or almost black, depending on the apple variety and the pathogen causing the disease. As the spots grow further, parasite fruiting bodies—acervuli—become visible on their surface (Figure 1b) whereby numerous slightly raised pustules radiate from the center of the spot to its perimeter. In most cases, the fruit tissue beneath the pustules darkens.

**Figure 3:**
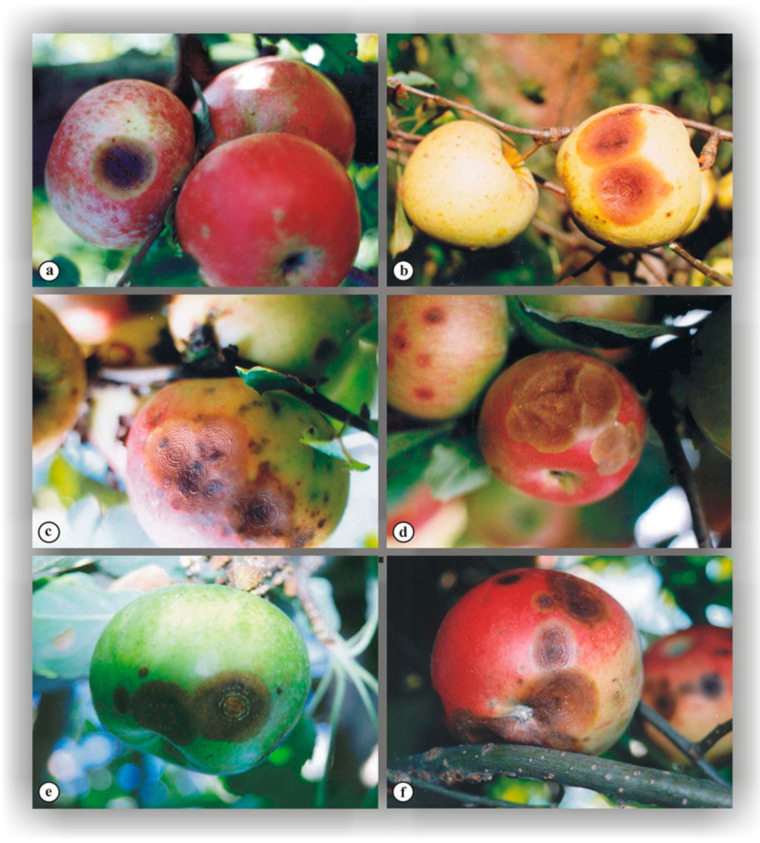
*Colletotrichum* spp. Bitter rot symptoms in apple orchard noted on the following cultivars: (**a**) Idared; (**b**) Golden Delicious; (**c**) Jonagold; (**d**) Red Jonathan; (**e**) Granny Smith; and (**f**) Prima (natural infection) (photo V. Trkulja).

In humid conditions, a large number of conidia develop from acervuli, forming a gelatinous mass of cream or yellowish-orange color (Figure 1f), whereas during dry weather, the conidial mass becomes more compact (Figure 2). Within the lesion, the fruiting bodies are often arranged in concentric circles (Figure 1b,d) commonly associated with the infection by this parasite. However, acervuli within the lesions can also be irregularly distributed (Figure 3b). The disease progression has been found to be primarily driven by the weather conditions, with warm and humid weather favoring rapid rot development and the formation of numerous acervular rings. This perspective is supported by similarity in the ABR symptoms (Figure 3a–f) among different apple varieties [12].

Empirical data further indicate that, as the spots age, they progressively deepen, and their surface becomes wrinkled and almost black in color. As spots gradually expand and merge, complete rotting (Figure 1c) and fruit mummification becomes inevitable. Although the affected fruits typically ripen prematurely and fall off (Figure 1d), in some cases, they remain attached to the branch throughout winter.

**Figure 4:**
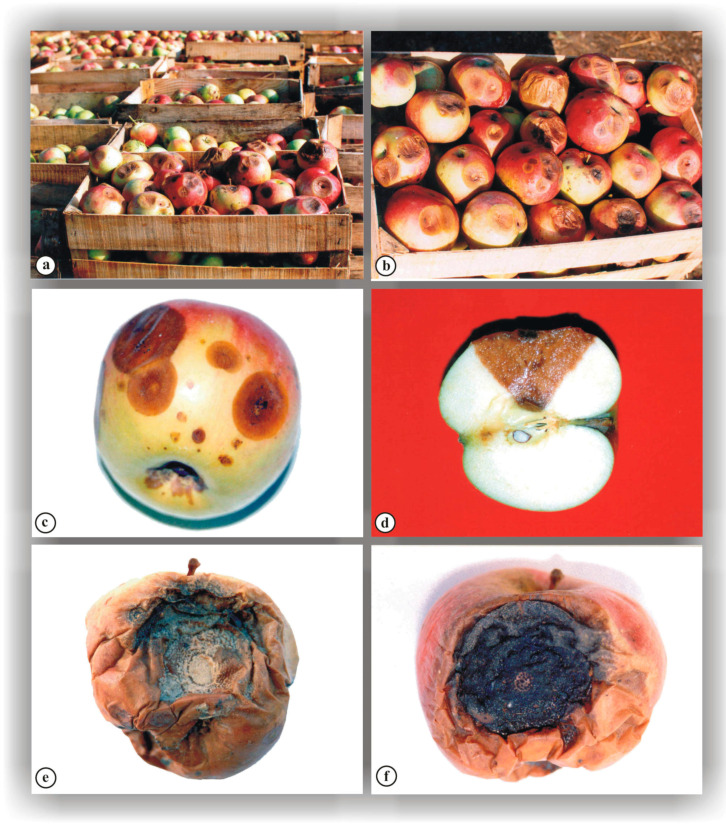
*Colletotrichum* spp. Bitter rot symptoms on apple fruits before and after removal from storage: (**a**) Apple fruits showing characteristic symptoms of bitter rot immediately after harvesting, before entering storage; (**b**) a more detailed view of harvested fruit [78]; (**c**) disease symptoms on apple fruit due to inadequate storage conditions; (**d**) characteristic tissue necrosis spreading towards the central part of the fruit forming a “V” shape [81]; (**e**,**f**) fruits affected by varying intensities of bitter rot with abundant parasite fruiting after removal from inadequate storage (photo V. Trkulja).

In infected fruit, changes also occur beneath the lesion surface, whereby flesh decay gradually progresses toward the core in the form of a cone, producing a characteristic V-shaped cross-section (Figure 4d), which is a characteristic symptom of this disease. According to Bompeix et al. [79] and Ivanović and Ivanović [80], in diseased tissue, the fungus produces toxins that give the infected fruit a bitter taste, which is why the disease was named “bitter rot”. However, Trkulja [12] established that, depending on the extent of disease progression, the flesh throughout the entire fruit may not always be bitter tasting, supporting Anderson’s [17] earlier observations.

Sometimes, fruits that had been infected in the orchard are placed in cold storage, where the parasite develops slowly due to low temperatures and causes less damage (Figure 4c). However, after removal from cold storage, and especially during sale in markets, the pathogen develops rapidly and causes the fruits to rot (Figure 4e,f). As a result, aided by other pathogens, particularly saprophytes, the disease spreads further [78,82].

In some cases, due to carelessness or ignorance, individual fruits with characteristic spots on which parasite sporulation has already occurred are brought into cold storage (Figure 4a,b), which is particularly dangerous, especially when storage conditions are inadequate, because it can lead to secondary infections and the development of small spots on healthy fruits in storage. The growth of these spots is slowed down due to low temperatures, which is why their outward appearance is usually different from the spots that form in the orchard. They develop very slowly in cold storage, but after bringing the fruits to the market they can spread very quickly (Figure 4e,f), thus causing significant economic damage [83,84]. The emergence and growth of small spots on healthy fruits in storage is slowed down due to low temperatures, which typically results in their different appearance compared to spots observed in orchards. These spots are usually small, red to purple with a dark center, closely resembling the physiological disorder Jonathan Spot Disease. Still, they can spread rapidly once fruits are removed from cold storage (Figure 4e,f), causing significant economic damage [83,84].

## 5. Symptoms of Apple *Glomerella* Leaf Spot

According to Taylor [65], GLS symptoms initially manifest as small red spots that, within about 10 days, develop into irregular yellow-brown lesions of 3–12 mm diameter. Under conditions conducive to the parasite’s development, these spots expand and merge, affecting entire leaves in some cases. When lesions coalesce due to severe infection, the affected leaf withers and falls off within two weeks, while less severe infections cause yellowing, followed by leaf fallout within 2–4 weeks. The disease in leaves that are fully mature at the time of infection gives rise to a wide range of symptoms, from small to dark brown necrotic spots, and may also cause leaf fallout. In resistant varieties, infected leaves tend to curl without visible necrosis. When high temperatures and low humidity persist during summer months, disease development is enhanced, and yellowing leaves tend to fall out in waves, leading to nearly complete defoliation of affected trees [65]. During their field studies, Shane and Sutton [85] similarly noted the emergence of brown spots, which quickly developed into large lesions during May and June. In a subsequent study conducted in Brazil on Gala and Golden Delicious varieties, Leite et al. [26] observed necrotic spots expanding into irregular areas reaching 3–10 mm in diameter. These authors also reported that, within 2–3 weeks after the initial symptom emergence, severely affected leaves became chlorotic and fell out. The authors also pointed out that in Gala, necrotic leaf spot tends to affect younger leaves, whereas in the Golden Delicious variety, middle shoots are typically the first and most severely affected. Similar symptoms were reported by Araújo and Stadnik [86], who stated that reddish-purple spots were noticeable on Gala leaves just two days after infection. Most of these spots later merged, giving rise to irregular necrotic lesions 7 to 10 days later, and the affected leaves turned yellow and eventually fell off. According to Sutton and Sanhueza [66], diseased fruits can also typically be found on infected trees, but the small, light brown, slightly sunken lesions (1–3 mm in diameter) do not increase in size over time.

Carvalho et al.’s work [87] focused on the *C. gloeosporioides* effects on Brazilian Gala orchards, where the authors recorded characteristic GLS symptoms on the leaves. The disease progressed rapidly post-inoculation, producing small red to light brown spots as early as two days later. In the infected leaves, lesions coalesced (covering almost the entire leaf surface) and became necrotic within three days, after which leaves started to wilt. Leaf losses became obvious within six days post-infection, leading to near complete defoliation of inoculated branches by day seven. On the other hand, *C. acutatum* isolates did not induce disease on Gala leaves [87]. According to the findings reported by Casanova et al. [88], in Uruguay, leaf spots caused by *C. gloeosporioides* are initially small (1–3 mm in diameter) and have a purple to brown color. They expand rapidly into larger irregularly-shaped necrotic lesions, demarcated by leaf veins. As the disease progresses, large sections or even entire leaves turn light brown to gray. Although lesions are visible on upper as well as lower leaf surfaces, acervuli and/or perithecia appear only on the edge of the upper surface, which takes on a dark color. Affected fruits can be distinguished by round and slightly sunken lesions typically with a red border, measuring 0.5 to 2 mm in depth, and 0.5 to 3 mm in diameter [88].

As a part of their work conducted in China, Wang et al. [89] documented severe damage to Gala and Golden Delicious varieties, where the disease caused defoliation in nearly all trees before the harvest. The symptoms resembled those of GLS observed in Brazil in 1988 and in the United States in 1998 on the same apple varieties. Initially, affected leaves developed small black lesions, which rapidly expanded (forming 2–3 cm-diameter circles with fuzzy margins) once the temperatures surpassed 30 °C, causing the leaves to darken and fall off. Although black lesions ceased spreading after 5–6 days at lower temperatures, large necrotic spots with clear margins nonetheless formed on affected leaves, causing them to turn yellow and fall off. As these black lesions produced numerous yellow conidia after 1–2 days of incubation at 30 °C and 100% relative humidity, these findings underscore the importance of these environmental factors in pathogen development and disease spread on apple leaves.

The most recent reports on GLS support the previously obtained findings. For example, Velho et al. [72] observed necrotic spots on Gala apple leaves in Santa Catarina, Brazil, in the summer of 2012 when the temperatures were at their peak. Within 7–10 days from the symptom onset, reddish-brown spots developed into necrotic lesions of 1–10 mm diameter. Casanova et al. [88] concurred with these findings, adding that these necrotic lesions tend to coalesce, especially on younger leaves, resulting in general chlorosis and defoliation.

As established by Sutton and Sanhueza [66], ascospores, which develop in perithecia on overwintering leaves, are the primary source of infection, while further disease spread is facilitated by perithecia that form in lesions on leaves in orchards, serving as secondary sources of infection. In nature, GLS isolates often form perithecia on apple leaves [71]. However, as perithecia have not been observed on infected fruits, isolates causing bitter rot in fruit appear to prefer asexual reproduction [68]. Research conducted by Carvalho et al. [87] in Brazil suggests that *C. gloeosporioides* isolates do not exhibit differences in pathogenicity between forms with and without perithecia. Moreover, Sutton and Sanhueza [66] confirmed that *G. cingulata* is capable of reproducing in numerous lesions on leaves that are still attached to the tree.

Wang et al.’s report from China [73] emphasizes the possibility of distinguishing between pathogens *C. fructicola* and *C. aenigma* based on their pathogenicity towards leaves and fruits of different apple varieties. Even though most *Colletotrichum* isolates cause either ABR or GLS, as established by Velho et al. [71], Børve and Stensvand [90] and Rockenbach et al. [91], some isolates can induce both diseases.

## 6. Geographical Distribution

As a causative agent of ABR, *Colletotrichum* spp. is one of the most significant threats to global apple production. Its first descriptions were provided by Berkeley in 1856 [31], who extensively studied this pathogen in England, but plant pathologists also documented its presence in the US at the end of the 19th century [23]. GLS was first described in apple by Leite et al. [26] in the state of Paraná, Brazil. However, evidence that accumulated over the years confirms that bitter rot causes substantial economic losses in all parts of the world where apples are grown, and apple leaf spot is most damaging in Asia, South America, and North America [92].

**Figure 5:**
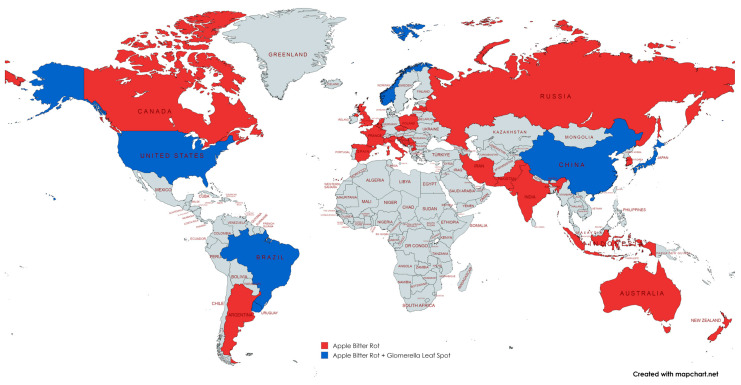
The world distribution map of apple diseases caused by *Colletotrichum* spp.: 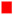 ABR only; 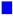 ABR + GLS (photo V. Trkulja).

In Europe, the occurrence of apple fruit diseases caused by *Colletotrichum* spp. has been documented in several countries. Although the first reports pertain to Russia [93], the fact that this pathogen is widely distributed is evident from subsequent studies conducted in Serbia [94,95], Bosnia and Herzegovina [77,83,84,96,97], Norway [98,99], Germany [100], Croatia [101], Italy [102,103,104,105,106,107,108], Czech Republic [109], the United Kingdom [110], Slovenia [111], Latvia [112,113], France [114,115], Netherlands [116], Belgium [117], North Macedonia [118], Spain [119], and Poland [120] (Figure 5).

In Asia, species of the genus *Colletotrichum* not only cause ABR but also induce GLS, as confirmed by research conducted in South Korea [121,122,123,124,125,126], China [7,127,128], Iran [129], India [130], Japan [131] and Pakistan [132] (Figure 5).

ABR and GLS are also present in North and South America. Both diseases have been identified across the US territory, including Alabama [133], Michigan [134], Arkansas and Virginia [135], North Carolina [136], Kentucky [137,138], Illinois [139,140], the Mid-Atlantic region [4,141] and New York State [5]. In Canada, the only evidence presently pertains to the Ontario province [142]. In South America, GLS and ABR have been identified in Brazil [68,71,76,136,143,144,145,146,147], Uruguay [71,88,147,148,149], and Argentina [150] (Figure 5).

While the evidence of ABR presence in New Zealand dates back to the 1970s [151], as confirmed by several subsequent investigations [152,153,154,155], this disease continues to affect apples in this country, whereas only one report currently pertains to Australia [156].

Although research on this topic is ongoing, the current distribution of *Colletotrichum* spp. is still not known with certainty. The earlier reports are particularly problematic, as they were based on morphology, pathogenicity tests, or the characterization of a limited number of genes. As none of these methods is sufficiently reliable for identifying *Colletotrichum* species, only findings yielded by molecular analyses (e.g., multigene phylogenetic analysis) should be relied upon when estimating the size and distribution of the global *Colletotrichum* spp. population.

## 7. Economic Importance

According to Anderson [17], prior to the development of effective fungicides, bitter rot was a highly dangerous disease, capable of destroying entire apple orchards within just a few weeks during warm and humid weather, as was the case in the 1880–1910 period. While such severe losses are relatively rare today, and are limited to relatively small areas under specific weather conditions, suboptimal storage parameters [83,84] and inappropriate handling upon removal from storage (i.e., during transport and sale) can still pose threats to the economic viability of apple harvests [78,82,157].

Despite its wide distribution, yield reduction due to ABR is much more pronounced in subtropical and tropical regions than in temperate climates, as its causative agents thrive at higher temperatures [79,158]. As a result, Bompeix et al. [79] posited that its practical importance in Europe is far less significant than in parts of North America, where summers are warmer and more humid [133,134,135], especially in the subtropical parts of this continent. However, modern transportation and storage technologies, combined with the overall improvements in modern apple cultivation systems and the development of effective control programs, have contributed to a marked decline in losses due to this disease since the 1950s.

Nonetheless, during the unusually warm and humid summers, even these advanced measures cannot counteract the natural disease cycle. For example, under such conditions, in apple orchards in Michigan, northeastern US, where ecological conditions for disease occurrence are generally unfavorable, 2–3% of fruits showed symptoms of bitter rot in 1995 [134]. In contrast, in the southeastern parts of the country, where temperatures during the apple growing season are typically higher than in northern states, Noe and Starkey [159] reported losses of up to 80% in unsprayed orchards due to this disease. Similarly, in North Carolina [85] and Illinois [140], bitter rot in apple orchards is usually first noticed during late June, and results in a complete yield loss in some years [85]. Therefore, it is not surprising that Taylor [160] considered ABR one of the most significant diseases in the southeastern United States more than five decades ago. Shane and Sutton [85] concurred with this view, adding that, as its causative agents have a short incubation period and rapidly sporulate on infected fruits, bitter rot can devastate entire orchards under favorable conditions. Even leaf infections can significantly reduce yields, but also contribute to overall tree weakening and up to 75% defoliation by harvest time under favorable conditions [68,69].

These findings highlight the exceptionally high economic importance of this leaf disease, given that even a single spot on an apple fruit typically precludes its sale [159]. According to Freeman et al. [1], further damage is caused by latent infections during storage.

## 8. *Colletotrichum* Species Causing Apple Bitter Rot

According to the currently available data, apple (*Malus* spp.) serves as host to 27 species of the genus *Colletotrichum*, the causative agent of ABR.

1. *Colletotrichum acutatum* J. H. Simmonds was first isolated and described by Simmonds [161] from diseased papaya (*Carica papaya* L.) tissue. However, this widely distributed species has since been recorded on various hosts [3]. To date, *C. acutatum* as the causative agent of ABR has been confirmed in the United States [134,135,136,139], Brazil [87], New Zealand [152,154,155], Korea [121], Indonesia [162], Uruguay [148] and Japan [163]. In Europe, its presence was initially noted in Bosnia and Herzegovina [12,84,96], and later in Norway [98,164], Italy [102], Czech Republic [109], Croatia [101], Latvia [112], and Belgium [117].

2. *Colletotrichum aenigma* B. S. Weir & P. R. Johnst. is a member of the *C. gloeosporioides* species complex. Its name derives from the Latin word *aenigma*, signifying its enigmatic nature in terms of biological and geographical distribution [10]. Its capacity to cause ABR has been identified in the United States [165] and South Korea [122].

3. *Colletotrichum alienum* B. S. Weir & P. R. Johnst. is another member of the *C. gloeosporioides* species complex, and owes its name to its biology and distribution on exotic hosts, such as those found only in Australia and New Zealand [10]. Thus far, this species has been identified as an apple pathogen in the United States [166] and China [7].

4. *Colletotrichum camelliae* Massee, belongs to the *C. gloeosporioides* species complex, and was first described by Massee [167] in living tea leaves [*Camellia sinensis* (L.) O. Kuntze] from Sri Lanka [10]. This species has been identified as an apple pathogen in the US [165].

5. *Colletotrichum chrysophilum* W. A. S. Vieira, W. G. Lima, M. P. S. Câmara & V. P. Doyle—as another member of the *C. gloeosporioides* species complex—was first described as a pathogen of banana [168]. It has been identified as the causative agent of ABR in the Mid-Atlantic region of the United States [4,6,141], where it has become the second most dominant species causing this disease [5]. In Europe, the presence of *C. chrysophilum* has been confirmed in Spain [119] and Italy [107], where it affected several apple orchards.

6. *Colletotrichum clavatum* Agosteo, Faedda & Cacciola, causing anthracnose of olive in Italy, was described as a new species within the *C. acutatum* species complex [169]. Since then, it has been identified as a pathogen of apple in Croatia [101].

7. *Colletotrichum conoides* Y.Z. Diao, C. Zhang, L. Cai & Xi L. Liu was described as a new species within the *C. gloeosporioides* complex, as the causative agent of anthracnose of a special chili pepper variety (*Capsicum annuum* var. *conoides*) in China, after which the species was named [170]. This species has been identified as an apple pathogen in the US [165].

8. *Colletotrichum fioriniae* (Marcelino & Gouli) Pennycook also belongs to the *C. acutatum* species complex. It derives its name from *C. acutatum* var. *fioriniae*, which was in turn named after a series of strains isolated from the insect *Fiorinia externa* Ferris [171]. It has a wide distribution and the capacity to infect a variety of hosts, including almonds, apples, avocados, mangoes, and nectarines [3,172]. Apple bitter rot caused by this species was first reported in Croatia [101], followed by the US [173], Slovenia [111], France [114], India [130], South Korea [124], Belgium [117] and Italy [103]. In the Mid-Atlantic region of the United States, it is recognized as the most aggressive and prevalent species causing ABR [4,5,137,141,165]. According to the evidence from different parts of the US, Khodadadi et al. [6], however, cautioned that it is also capable of causing bitter rot in apple fruit during storage.

9. *Colletotrichum fructicola* Prihast., L. Cai & K. D. Hyde exhibits the widest geographic diversity within the *C. gloeosporioides* complex [10]. Initially, it was described by Prihastuti et al. [174] as a pathogen of coffee in Thailand. It has since been identified as the causative agent of ABR in China [127,175], Iran [129], Brazil [71,75,91,144], Uruguay [71,75,91,149], the United States [166], and Japan [131]. While in Japan this pathogen is recognized as the most common and aggressive species causing this disease, it has also been documented in apple orchards in India [130], France [115], and Italy [106].

10. *Colletotrichum gloeosporioides* (Penz.) Penz. & Sacc. [teleomorph: *Glomerella cingulata* (Stoneman) Spauld. & H. Schrenk] is a widely distributed species with numerous hosts [10]. It has been confirmed as a pathogen of apple in the United States [4,135,136,141,166], Bosnia and Herzegovina [12,83,84], Brazil [87,176], New Zealand [152], Korea [121,123], Uruguay [148], Latvia [112], and China [7]. The teleomorph stage of this fungus, *Glomerella cingulata*, has also been identified as a causal agent of ABR in the United States [65,69,85,133,135,136,159,165,177], New Zealand [151], and Brazil [68,178].

11. *Colletotrichum godetiae* Neerg., belonging to the *C. acutatum* species complex, was first isolated and described by Neergaard in 1943 after isolating it from *Clarkia amoena* (syn. *Godetia amoena*) cv. Kelvedon Glory seeds [3]. This pathogen occurs on various hosts—including genera *Malus*, *Prunus*, and *Fragaria*—primarily in Europe and the Middle East [3]. Thus far, it has been confirmed as a pathogen of apple in the United Kingdom [110], Slovenia [111], Latvia [113], the Netherlands [116], Belgium [117], Italy [103], and Canada (Ontario) [142]. In Belgium, it is recognized as the most pathogenic causative agent of ABR. Moreover, pathogenicity tests confirmed that its isolates from strawberries can also cause disease on apple fruits [117]. 

12. *Colletotrichum grevilleae* F. Liu, Damm, L. Cai & Crous is a member of the *C. gloeosporioides* species complex, and was described by Liu et al. [179] as a causal agent of root and collar rot in *Grevillea* species in Italy, from which its name derives. It was recently identified on Fuji apple fruits in South Korea [126].

13. *Colletotrichum grossi* Y.Z. Diao, C. Zhang, L. Cai & X.L. Liu (as “grossum”)—another member of the *C. gloeosporioides* species complex—derives its name from *Capsicum annuum* var. *grossum* (Willd.) Sendtn., as it was originally identified in bell pepper in China [170]. It was recently also identified in Italy as a causal agent of bitter rot in apple [108].

14. *Colletotrichum henanense* F. Liu & L. Cai also belongs to the *C. gloeosporioides* species complex, and is named after Henan Province in China, where it was originally identified. This species was first described as a pathogen of *Camellia sinensis* (L.) Kuntze and *Cirsium japonicum* DC. in China [180], and has subsequently been confirmed as a pathogen of apple in the US [4,141,165].

15. *Colletotrichum kahawae* J. M. Waller & Bridge—another member of the *C. gloeosporioides* species complex—has been described as a pathogen of coffee berries in several African countries [10,181]. Thus far, its capacity to infect apple has been confirmed in Belgium [117] and the US [138,165].

16. *Colletotrichum limetticola* (R. E. Clausen) Damm, P. F. Cannon & Crous belongs to the *C. acutatum* species complex. Its name derives from lime (*Citrus aurantifolia* (Christm.) Swingle) leaves and twigs in Cuba, where it was first identified and described as *Gloeosporium limetticola* by Clausen in 1912 [3]. In Brazil, isolates obtained from apple flowers have been shown to cause symptoms on apple fruits as well [146].

17. *Colletotrichum melonis* Damm, P.F. Cannon & Crous also belongs to the *C. acutatum* species complex. This species was described by Damm et al. [3] as an isolate originally from Brazil, obtained from melon (*Cucumis melo* L.) rind, from which it derives its name. To date, it has been identified on apple fruits in Uruguay [71,75,149] and Brazil [145].

18. *Colletotrichum noveboracense* F. Khodadadi, P.L. Martin, V.P. Doyle, J.B. Gonzalez & S.G. Aćimović—named after the Latin term “*Noveboracum*” for New York, emphasizing its origin—was described by Khodadadi et al. [5]. This pathogen of apple has been identified across the Mid-Atlantic region of the US [4,5,6,141].

19. *Colletotrichum nupharicola* D. A. Johnson, Carris & J. D. Rogers—member of the *C. gloeosporioides* species complex [10]—was initially confirmed in the United States as a pathogen of water lilies (*Nuphar* species), from which it derives its name [182]. Its presence on apple fruits in the US has since also been confirmed [6,165].

20. *Colletotrichum nymphaeae* (Pass.) has a wide range of host plants [3]. This member of the *C. acutatum* species complex was initially identified on water lily (*Nymphaea alba* L.), from which it derives its name [182]. It is recognized as the most common pathogen on apple fruits in Brazil [145], based on several reports [71,75,143], but is also found in India [130], Korea [124], the US [141,165], and China [7].

21. *Colletotrichum orientale* Dandan Fu & G.Y. Sun (as “orientalis”) also belongs to the *C. acutatum* species complex and was first reported in China as a causal agent of ABR [7].

22. *Colletotrichum paranaense* C.A.D. Bragança & Damm—another member of the *C. acutatum* species complex [57]—was first identified and described as a pathogen of apple and peach fruits in the state of Paraná, Brazil, from which it derives its name [145]. It has since also been identified as a causal agent of ABR in Uruguay [75].

23. *Colletotrichum rhombiforme* Damm, P. F. Cannon & Crous is characterized by its rhomboid ascospores, from which it derives its name [3]. This *C. acutatum* species complex member has been identified as a causal agent of ABR in China [183] and Belgium [117].

24. *Colletotrichum salicis* (Auersw. ex Fuckel) Damm, P. F. Cannon & Crous—yet another member of the *C. acutatum* species complex—has wide global distribution, with reports of its presence on several hosts in the US, New Zealand, Germany, the Netherlands, and Iran [3,129]. Thus far, it has been identified as a causal agent of ABR in Belgium [117] and Italy [105].

25. *Colletotrichum siamense* Prihast., L. Cai & K. D. Hyde belongs to the *C. gloeosporioides* species complex [10]. First described as a pathogen of *Coffea arabica* L. berries [174], this species is capable of infecting numerous hosts, including apple [10]. To date, it has been reported to cause ABR in Japan [131], the United States [4,165,166], Argentina [150], South Korea [124,125], Pakistan [132], and China [7,184]. In the US [137] and India [130], it is recognized as the most aggressive species causing bitter rot in apple fruit.

26. *Colletotrichum theobromicola* Delacr.—another member of the *C. gloeosporioides* species complex—is widely distributed and affects numerous hosts in tropical and subtropical regions [10]. To date, it has been identified as a causal agent of ABR in Uruguay [71,75,149], India [130], and the US [137,165].

27. *Colletotrichum tropicale* E.I. Rojas, S.A. Rehner & Samuels also belongs to the *C. gloeosporioides* species complex [10]. Its name derives from various host plants from the tropical forests of Panama [185]. In North Carolina, eastern United States, this species caused considerable damage to apple orchards in 2014 and 2015, despite not previously being recognized as a pathogen of this fruit species [166].

## 9. *Colletotrichum* Species Causing *Glomerella* Leaf Spot in Apple

Various species of the genus *Colletotrichum* are known to cause damage to apple leaves. According to the available data, the following 15 species of fungi from this genus have been identified worldwide as causative agents of GLS:

1. *Colletotrichum acutatum* was identified on apple leaves in Norway [186];

2. *Colletotrichum aenigma* as a GLS causative agent has been confirmed in China [7,73,128] and Japan [131];

3. *Colletotrichum alienum* has been identified as the cause of apple leaf spot in the US [166];

4. *Colletotrichum asianum* Prihast., L. Cai & K. D. Hyde was initially described by Prihastuti et al. [174] on coffee berries (*Coffea arabica* L.). Since then, this member of the *C. gloeosporioides* species complex [10] has been confirmed on apple leaves in China [74];

5. *Colletotrichum chrysophilum* has been confirmed as a GLS causative agent in Brazil and Uruguay [147];

6. *Colletotrichum fioriniae* has been identified as a pathogen of apple leaves in the United States [187];

7. *Colletotrichum fructicola* has been reported as the cause of apple leaf spot in China [7,73,92], Brazil [72,75,76,91,144], Japan [131], the US [166], and Uruguay [88,188];

8. *Colletotrichum gloeosporioides* (teleomorph: *Glomerella cingulata*) has been identified as the cause of apple leaf spot in Brazil [87] and the US [166]. The presence of its teleomorph stage, *G. cingulata*, has been reported in the United States [65], Brazil [67,68,69,136], and China [89]. However, two isolates of the fungus (ICMP 17787 and ICMP 17788) from Brazil, identified in 2001 by T. Sutton as *C. gloeosporioides*, were later reclassified as *C. fructicola* by Weir et al. [10];

9. *Colletotrichum karsti* You L. Yang, Zuo Y. Liu, K.D. Hyde & L. Cai (as “karstii”) was described by Yang et al. [189] in China on the leaf of the orchid *Vanda* sp. (*Orchidaceae*). It occurs on a large number of hosts and is the most geographically widespread species within the *C. boninense* species complex [9]. It has been detected on apple leaves in Brazil [70,71,75] and Uruguay [188];

10. *Colletotrichum limetticola* has been identified as the causative agent of apple leaf spot in Brazil [76,146];

11. *Colletotrichum melonis* has been identified as the causative agent of GLS in the Brazilian states of São Paulo and Paraná [76];

12. *Colletotrichum nymphaeae* has been identified as the cause of apple leaf spot in Brazil [72,76];

13. *Colletotrichum paranaense* has also been identified as a pathogen of apple leaves in Brazil [76];

14. *Colletotrichum siamense* has been identified as the causative agent of apple leaf spot in Japan [131] and the United States [166];

15. *Colletotrichum tropicale* has been detected on apple leaves in North Carolina, US [166]. However, in this study, *C. tropicale* was not confirmed as a direct pathogen of apple leaves, so additional research is needed to confirm its pathogenicity on apple leaves.

## 10. Disease Cycle

Species of the genus *Colletotrichum* that are recognized as pathogens of apple fruits and leaves can survive from one growing season to another. They persist in infected buds, mummified apple fruits on the tree, infected branches and twigs in the woody parts, and cankers. They also survive in fallen leaves that were infected during the previous growing season, providing a source of primary inoculum for the next growing season [4,13,29,65,98,146,155,160,165,190,191,192,193,194,195,196,197,198]. According to the studies conducted by Hamada and De Mio [195] in Brazil, infected fallen leaves, along with dormant buds and infected twigs, serve as the most common sources of inoculum for the next season. These findings concur with those reported by Taylor [65] and Sutton [13] for the US, and Børve and Stensvand [98] for Norway. Based on the research conducted in southern Brazil, Crusius et al. [193] noted that the pathogen is only capable of asymptomatic survival in dormant buds and twigs, but not in fallen apple leaves on the ground or in mummified fruits. However, Sutton [13] more recently established that fruit remnants following chemical tree treatment, as well as mummified fruits on the ground, are potentially significant sources of *C. gloeosporioides* inoculum for the upcoming growing season. Taylor [160] was among the first authors to highlight the role of mummified fruits as a primary source of inoculum for fruit infection, either before, during, or shortly after flowering. On the other hand, Nekoduka et al. [163] posited that infected fruit scars are the primary source of inoculum, suggesting that the pathogen can asymptomatically persist in apple flowers during the growing season. Such latent infection is crucial for further spread to immature fruits, as later confirmed by Hamada et al. [146]. In New Zealand, buds are a more significant source of primary inoculum for *C. acutatum* than infected branches [155]. As a part of the same study, the asymptomatic infection of vegetative and reproductive buds was also confirmed. *C. acutatum* has also been found on asymptomatic surface-sterilized petals and fruits, more frequently during summer than in spring.

**Figure 6:**
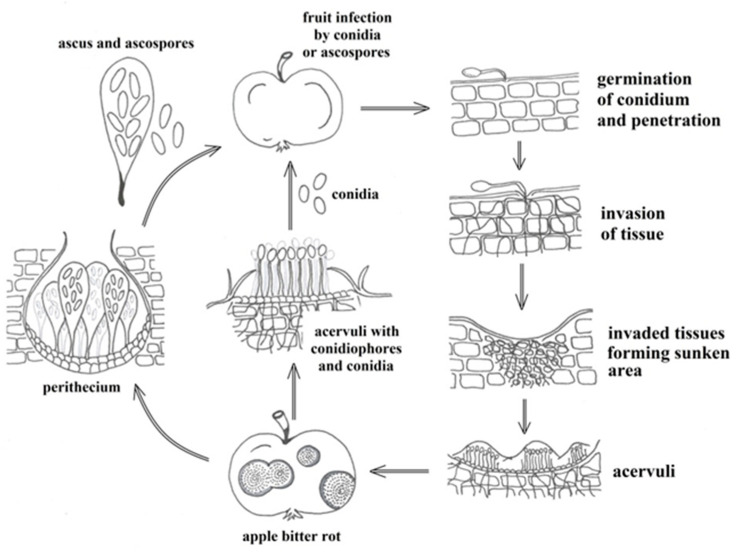
*Colletotrichum* spp. disease cycle of apple bitter rot (photo T. Popović Milovanović).

The developmental cycle of pathogens causing fruit anthracnose in apple comprises a sexual stage (which includes the formation of perithecia with asci and ascospores) and an asexual stage (during which the pathogen forms acervuli with conidia) (Figure 6). Pathogens can serve as the primary source of inoculum for new infections in either of those stages. Nonetheless, several authors argue that conidia and ascospores produced during the previous growing season are the main contributors to primary infections in spring [65,194,196]. During the growing season, the pathogen can also cause several secondary infections, as conidia and ascospores are easily spread by raindrops and wind [4,11,13,151,155,165,177,190,191,192,194,196,198,199,200,201,202]. As shown by Sutton and Shane [177], in apple orchards in the US, ascospore release typically commences after 2–3 h of persistent rain, and their number is the highest during rainy periods that coincide with the growing season. Using traps, these authors also established that conidia are far less abundant than ascospores, and are typically present from May to early June [177]. In Brazil, conidia were recorded on infected trees from October to May, from which they also tended to wash off, as indicated by the presence of spores in the air from October to the end of June. The airborne spores were most abundant in January and February, whereby the highest quantities of conidia were captured in traps elevated 30 cm from the ground [202]. Insects, birds, and humans, as well as tools and agricultural machinery used in orchards, also play an important role in spreading conidia during the growing season [190,194,198,200,202].

After the winter dormancy period, if humidity and temperatures are at a moderate level in the spring, the pathogen causes initial infections on flowers and leaves, later progressing to fruits, potentially leading to secondary infections that persist until harvest [165,196]. In orchards, primary leaf infections are most common in the lower parts of the canopy, allowing the formation of new secondary inoculum, and spread to the upper canopy parts under favorable environmental conditions [198]. The fungus spreads rapidly owing to its ability to produce abundant conidia in acervuli and ascospores in perithecia within infected (necrotic) lesions [151,188,194,198], as well as its capacity to continually form reproductive structures on the upper leaf surface [11,65,88,190]. Consequently, serious infections are more likely to occur in years with above-average rainfall due to increased inoculum production during the growing season. As established by Leonberger et al. [165], warmer weather favors acervuli formation on infected tissue, whereas moisture facilitates water absorption and conidia release, which is sometimes accompanied by the appearance of orange-colored exudate containing pathogen spores [165]. Jones et al. [134] also detected orange-colored exudate containing acervuli and conidia on lesions in inoculated apple fruits (Figure 6).

## 11. Epidemiology

As infected parts of the canopy such as buds and branches (twigs), as well as mummified fruits and fallen leaves, are the most significant sources of primary inoculum for the next growing season, when environmental conditions are favorable (higher temperatures and humidity), the disease severity tends to increase [70,155,190,191,200,201]. According to the empirical evidence, under favorable environmental conditions, sporulation occurs in about 10 days after the infection.

As the pathogen penetrates through wounds or directly into the tissue, it can be quickly colonized, after which the infection spreads both inter- and intracellularly [11,194]. The lifestyles of *Colletotrichum* species can be broadly classified as necrotrophic, hemibiotrophic, latent, and endophytic [203], whereas according to their colonization strategies, they are categorized as either intracellular hemibiotrophs (IHB) or subcuticular-intramatrical necrotrophs (SIN). In IHB colonization, infective hyphae penetrate epidermal cells, whereas in SIN, the fungus grows within the periclinal and anticlinal walls of epidermal cells beneath the cuticle [198,203]. Although most *Colletotrichum* species colonize tissues via IHB, SIN colonization or a combination of both strategies has been recorded for some species [198,203,204]. In the SIN strategy, the interaction is primarily necrotrophic, whereas the biotrophic phase is either very short or nonexistent. As disease symptoms caused by *C. gloeosporioides* typically emerge 45 h after inoculation, this points to SIN as the primary apple leaf colonization strategy [198,200,203,204].

In most cases, infection occurs from the mid to the late season, but also during or immediately after flowering [13,194]. Although fruits remain susceptible to infection throughout all stages of development, they are particularly at risk three weeks after petal fallout until harvest [194]. Infected fruits, as well as mummified fruits that remain on branches following chemical treatment, serve as additional sources of inoculum during the growing season [13,194]. Severe disease outbreaks typically occur when summer and fall are warm and humid, but have also been recorded after a primary infection early in spring, due to the presence of abundant secondary inocula [13,151,190].

During infection after spore germination, pathogenic species of the genus *Colletotrichum* always form an appressorium. However, daily temperatures need to exceed 15 °C for the overwintering forms of the pathogen to form conidia and subsequently an appressorium [153,201,205]. Even when the pathogen’s reproductive organs (conidia and/or ascospores) enter the plant organ, infection will only occur under optimal environmental conditions, i.e., sufficiently prolonged high temperatures and humidity [155,200].

Conidia and ascospores of *Colletotrichum* species can germinate and develop appressoria in water droplets, due to which fruit can be infected within 5 h if the temperature is around 26 °C [13]. According to Moreira et al. [203], who studied the germination of the *C. acutatum* species complex (*C. nymphae*, *C. paranaense*, and *C. melonis*) and *C. gloeosporioides* species complex (*C. fructicola*, *C. siamense*) under laboratory conditions on artificial PDA media, their optimal temperatures for germination are 15–25 °C and 20–25 °C, respectively. These authors further observed that conidia of both complexes germinated within 6 h after inoculating detached apple leaves and fruits (wounded and unwounded), and appressoria formed within 24 h after inoculation in all cases except on unwounded fruits. Wang et al. [201] similarly established that *C. gloeosporioides* conidia germinated and formed appressoria on inoculated apple leaves at 15–35 °C. While 27.6 °C was the most optimal temperature, for germination to occur, air humidity had to exceed 99% or leaves had to be covered by water droplets. Crusius et al. [193], however, highlighted the importance of leaf wetness duration for infection, ranging from 2–4 h at 24–30 °C to 32 h at 16 °C. The authors also noted that infection was inhibited outside the 14–34 °C temperature range. These findings supplement those reported by Sutton et al. [13], according to whom leaf wetness must persist for at least 16 h for infection to occur at 16–24 °C.

Wang et al. [201] similarly noted the highest number of lesions resulting in overall infection at 25 °C, whereas outside the 15–30 °C temperature range, only a small number of conidia successfully infected leaves, which subsequently developed symptoms. According to Nita et al. [205], 20–30 °C is the optimal temperature range for lesion development on apple fruits, depending on the pathogen species and cultivar. Similar conclusions were reached by Velho et al. [70], who found that high humidity combined with temperatures in the 23–28 °C range resulted in greater defoliation and disease severity. In an earlier study, Sutton and Shane [177] established that more pronounced rot symptoms occurred in fruits infected with *C. gloeosporioides* at 16–28 °C compared to temperatures below 12 °C and above 32 °C. Thus, Katsurayama et al. [200] concluded that disease development exhibits an upward trend as the temperature and humidity increase. Ellis [194] concurred with this view, stating that optimal temperatures for disease development range from 26 to 32 °C. Higher optimal temperatures for the development of the *C. gloeosporioides* species complex compared to the *C. acutatum* species complex were noted by Aćimović et al. [4]. In an earlier study, Everett et al. [155] similarly found that, even under humid conditions, for the infection of apple fruits with *C. acutatum* and the appearance of lesions to occur, the temperature must remain above 15 °C for at least 72 h. As previously noted, Moreira et al. [203] established that *C. gloeosporioides* species (*C. fructicola* and *C. siamense*) developed significantly larger colonies at 25 °C on PDA medium than *C. acutatum* complex species (*C. nymphaeae*, *C. paranaense*, and *C. melonis*), which required lower temperatures for optimal growth [203]. Nita et al. [205] similarly established that the optimal temperature for the fastest growth of *C. siamense* mycelium on PDA was between 25 and 30 °C, while *C. fioriniae* grew faster at 25 °C and slower at 30 °C. These results are supported by Velho et al. [70], according to whom the optimal temperature range for the development of species belonging to *C. gloeosporioides* on PDA medium was 22–24 °C (23.8 °C on average) and between 25 and 26 °C (25.6 °C on average) for species belonging to *C. acutatum*. In a subsequent study, Velho et al. [71] noted the maximum growth of *C. fructicola*, *C. karstii*, *C. nymphaeae*, *C. theobromicola* and *C. meloni* isolates at 25 °C. Grahovac et al. [95] previously reported similar findings, stating that *C. gloeosporioides* isolates grew above 32 °C, whereas *C. acutatum* isolates did not. Nonetheless, the optimal temperature range for both pathogens was 23–28 °C. The better growth of *C. cingulata* and *C. gloeosporioides* at 30 °C compared to *C. acutatum* was also recorded by González and Sutton [206].

Nearly five decades ago, Brook [151] observed that, following artificial inoculation at 21 °C and high relative humidity, conidia germinated within 2 h, forming germ tubes, whereby appressoria developed within the next 60 min. During the following hour, the appressoria grew to their final size (measuring 5–6 μm in diameter) and had thickened walls. Five hours after the inoculation, the appressoria became rounded and separated from the parent conidia, and over the next hour, their walls became thick and yellow. Once they attached to the apple surface, they began darkening, turning olive and finally brownish-black over the next few hours. On Day 2, penetration hyphae (measuring cca. 0.5 μm in diameter) passed through the basal pores of the appressoria, gaining direct access to the cuticles of the epidermal cells below. By Day 3, the contents of the epidermal cells beneath the appressoria turned brown. At sites where intercellular hyphae developed, necrotic lesions were evident by Day 7, while by Day 11 the authors observed sporulation and acervuli development within these necrotic lesions. These results were later reproduced under laboratory conditions by Wang et al. [201], who further noted that the shortest incubation period of 2 days was achieved at 25 °C and humidity above 99%. As a part of an earlier investigation, Katsurayama et al. [200] recorded a shorter incubation period in infected apple leaves compared to fruits, with symptoms appearing after 45 h and 96 h, respectively. Sutton and Shane’s [177] laboratory experiments on inoculated apple fruits also indicated that 24–28 °C was the optimal temperature range for the sporulation of all tested isolates. These authors also noted significantly reduced sporulation at 36 °C. According to their findings regarding artificial leaf inoculation, the sporulation of *C. acutatum* isolates was positively correlated with temperature up to 30 °C, while the rate was optimal at 25–30 °C. Based on their more recent study, Everett et al. [155] concluded that pathogen sporulation (conidia) in lesions required temperatures above 15 °C. Hamada et al. [146] also inoculated apple leaves and fruits with *C. acutatum* isolates as a part of their study, reporting that the first symptoms appeared on leaves after an incubation period of 3–5 days, while a 4-day incubation period was noted for injured fruits, extending to 14–15 days for uninjured fruits. Similar results were reported by Moreira et al. [203], highlighting that the incubation period for artificially inoculated injured apple fruits with species from the *C. gloeosporioides* and *C. acutatum* complexes ranged from 2 to 6 days, and from 5 to 25 days for uninjured fruits depending on the variety. Alaniz et al. [188] reported a 21-day incubation period after inoculation of apple fruits with *C. fructicola* and *C. karstii* at 25 °C and 100% relative humidity. On the other hand, under the same conditions, initial symptoms on separate leaves and shoot leaves appeared 3–7 days after inoculation. After testing *C. fructicola*, *C. karstii*, *C. nymphaeae*, *C. theobromicola*, and *C. melonis*, Velho et al. [71] recorded a shorter incubation period of 3–4 days on damaged fruits, 5–6 days on undamaged fruits, and 2–3 days on inoculated apple leaves for all pathogens. In the experiments conducted by Moreira et al. [76] involving the artificial inoculation of apple plant leaves in pots that were kept moist, at temperatures above 20 °C, the incubation period lasted between two (*C. nymphaeae*, *C. limetticola*, and *C. fructicola*) and four (*C. melonis* the longest) days, with a latent period of 8–11 days depending on the isolate. In their later study, Moreira et al. [203] confirmed these findings, noting a latent period of 9–12 days for *C. fructicola*.

## 12. Molecular Characterization

As accurate pathogen identification is crucial for the development of effective disease management strategies, to overcome the challenges associated with the genus *Colletotrichum*, traditional methods started to be combined with the molecular approaches in the last few decades. Contemporary studies tend to rely on polymerase chain reaction (PCR), RAPD fingerprinting, analysis of the internal transcribed spacer (ITS) region of rDNA sequences, and simultaneous sequencing of multiple genes. In these investigations, the final identification is achieved through the analysis of a phylogenetic tree obtained by sequencing multiple loci.

When traditional methods are employed, the identification of *Colletotrichum* species is based on various criteria, such as morphology, optimal growth temperature, vegetative compatibility, and response to benomyl, and these findings are usually supplemented with molecular investigations [1]. Although these strategies are beneficial for establishing values of certain parameters, such as the shape and size of conidia, perithecia production [1,207], growth rate on PDA [52], and benomyl sensitivity [1], they are insufficient for capturing variations due to environmental influences [208]. The insufficiency of morphological characteristics for accurate species differentiation within the complex was confirmed by Oo et al. [124], who identified new species through a combination of morphological and molecular characteristics. Genetic differences among *Glomerella cingulata* strains associated with GLS and ABR and those associated solely with ABR were also highlighted by vegetative compatible groups (VCG) analysis. However, as noted by González [69], the extent of these genetic differences cannot be determined via VCG. Thus, as a part the later study conducted by this author and colleagues, VCG was supplemented by morphological and molecular characterization methods, allowing them to successfully differentiate isolates pathogenic to leaves and fruits from those pathogenic to fruits only [68]. This comprehensive strategy allowed for a deeper understanding of the genetic and phenotypic variability among the studied isolates.

*Colletotrichum* species are frequently identified via ITS analysis [207]. This approach involves comparison of the sequences of the internal transcribed spacer of ribosomal DNA (ITS1-5.8S-ITS2 = ITS). This region has proven effective for distinguishing *Colletotrichum* species at the complex level [2,56]. However, differentiation at lower levels within the complex requires multigene sequencing [2]. The application of this advanced method has led to the separation of *C. gloeosporioides* and *C. acutatum* sensu lato into a greater number of individual species [3,10].

As a part of their study, Vieira et al. [209] analyzed various markers to identify the characteristics required for the differentiation of all *Colletotrichum* fungal species. While this goal was not attained, as markers effective for one species complex were not equally useful for other complexes, the authors concluded that a combination of three different genes could be useful for reliably distinguishing most species complexes. Accordingly, they suggested using one gene, such as *glyceraldehyde-3-phosphate dehydrogenase* (GAPDH) or *β-tubulin* (TUB2), for the initial determination of the complex, while relying on other genes (selected according to the complex characteristics of those species) for further analysis [209].

Although morphological and phylogenetic analyses based on ITS are not always sufficient for identifying *Colletotrichum* species at the species level, these challenges can be overcome by conducting phylogenetic tree analyses of multiple loci [5]. When this strategy is adopted for identifying species from the genus *Colletotrichum*, in addition to the ITS region, GAPDH, TUB2, *actin* (ACT), *chitin synthase* (CHS-1), *histone H3* (HIS3), *calmodulin* (CAL), *glutamine synthetase* (GS), *Mat 1-2 gene* (ApMat), and *DNA ligase* (APN2) are the most frequently used genes (Table 1). For ITS amplification, appropriate primer pairs are required, such as ITS1, ITS4, and ITS5 [210], along with specific primers for *C. acutatum* (CaInt2) and *C. gloeosporioides* (CgInt) [211]. For GAPDH, primers GDF1 and GDR1 [172] are in use, while primers Bt2a and Bt2b [212], T1 and T2 [213], BT2Fd and BT4R [214], and TB5 and TB6 [215] are typically adopted for TUB2. For ACT and CHS-1, the primers ACT-512F/ACT-783R and CHS-79F/CHS-345R are used, respectively [216]. CYLH3F and CYLH3R serve as primer pairs for the H3 gene [217], while primers CL1C and CL2C are used for the CAL gene [10]. For the GS gene, primers GSF and GSR [218] are used, primers CgDL-F6 and CgMAT1F2 are adopted for ApMat, and for APN2, primers ColDL-F3 and CgDL-R1 are typically chosen [185].

Analyses of various gene loci have proven particularly useful for more precise species differentiation and identification. For example, Lee et al. [121] conducted an analysis of isolates using a combination of random amplification of polymorphic DNA (RAPD), ITS rDNA sequencing, and partial TUB2 gene sequencing. The authors amplified rDNA–ITS and partial TUB2 genes using ITS1 and ITS4, as well as TI and βt2b, as primers, while relying on the PELF/URPIF primer pair for RAPD analysis. Based on the evaluation of molecular characteristics, they established that *C. acutatum* isolates from apple were clearly distinguished from red pepper isolates of the same species, whereas apple isolates of *C. gloeosporioides* were not [121]. More recently, Velho et al. [70] used a combination of the ITS region with primers ITS1/ITS4 and the GAPDH gene with primers GDF1/GDR1, indicating that the isolate sequences exhibited 100% homology with *C. nymphaeae*. The same gene combination (ITS/GAPDH) and the same universal primers were used by Oo et al. [124] to demonstrate the alignment of isolates with sequences belonging to different species from the genus *Colletotrichum*. As a part of their work, Alaniz et al. [149] identified four species based on the TUB2 gene and the ITS/GAPDH gene combination. In an earlier study, Alaniz et al. [148] relied solely on the ITS region. While they identified *C. acutatum* and *C. gloeosporioides*, by combining additional genes with ITS, these isolates were later re-identified as *Colletotrichum* sp., *C. melonis*, and *C. fructicola* [149]. These disparities in the results obtained even by the same groups of authors confirm that using only the ITS region is insufficient for determining differences at the species level. Fu et al. [127] identified *C. fructicola* based on a phylogenetic analysis of combined datasets (ITS/ACT/TUB2/GAPDH) and morphological characteristics of the anamorph. These authors used the following primer pairs for the amplification of gene regions: ACT-512F + ACT-783R, CHS-354R + CHS-79F, GDF1 + GDR1, CYLH3F + CYLH3R, BT2Fd + BT4R, and ITS1 + ITS4. More recently, Khodadadi et al. [5] used three genes (ITS, TUB2, and GAPDH) for species identification within the *C. acutatum* complex.

Understanding the biology of *Colletotrichum* spp. and the interactions of these pathogens with host plants is crucial for developing effective control strategies. Recent studies reveal significant differences in gene expression and biological characteristics among different isolates, especially those associated with GLS and ABR [220,221]. Available evidence also indicates that conidial anastomosis tube (CAT) and gene expression analyses are required to gain deeper insight into the biology of *Colletotrichum* spp. and their complex interactions with host plants. For example, the findings reported by Gonçalves et al. [220] highlight variations in the CAT development among different strains and their association with different *Colletotrichum* species, such as *C. fructicola* and *C. theobromicola*. These authors also discovered the potential for genetic variability through nuclear transfer in CATs. Jiang et al. [221] similarly noted significant differences in the expression of genes related to pathogenicity between isolates causing GLS and those causing ABR. The former demonstrated increased penetration ability and pathogenicity compared to the latter, indicating evolutionary changes through mutations into more virulent strains. These findings are essential for the developing effective control strategies for these pathogens, underscoring the need for continued research in this domain.

In sum, combining molecular analyses with biological characteristics assessments provides a more nuanced picture of the complex interactions between pathogens and host plants, paving the way for innovative approaches in plant disease management.

## 13. Control

ABR and GLS in apple can only be adequately controlled through an integrated program of cultural practices, combining sanitary, agrotechnical, and biological measures, as well as by cultivating resistant varieties while using chemical protection measures only when absolutely necessary [81,165,194,222].

### 13.1. Sanitation

Orchard sanitation programs encompass a variety of measures aimed at reducing potential sources of infection, such as removing mummified fruits and cankers, which may contain overwintering conidia and thus serve as the inoculum source [223]. Although infected fruits on apple branches are also sources of infection, infected rotten fruits that have fallen to the ground are particularly dangerous, as they contain spores that survive on the fruit surface and within soil during the autumn season. As they tend to overwinter successfully, they serve as a potent source of infection for healthy fruits in the next season. Likewise, apples that remain on the branches shrivel and overwinter in a mummified state, allowing spores within them to infect healthy fruits in the following season. To prevent this adverse outcome, all dried fruits on branches and rotten fruits on the ground should be removed [224]. In orchards affected by GLS, fallen apple leaves should be shredded in autumn with a mower to minimize the risk of infection transmission [68].

As cankers found on apple trees (typically those that are weakened and/or injured) are one of the most important sources of infection, they should be removed or burned [194]. Removing infected branches also helps reduce the presence of pathogens [225].

Given that piles of branches left on the soil surface after pruning can also serve as a source of infection, they should also be removed from the orchard as soon as possible [194].

### 13.2. Cultural Practices

These practices involve establishing proper plant spacing, regular pruning to improve air circulation [202], and weed management [165]. When combined with fungicide application, these measures can significantly reduce the impact of bitter rot [165]. However, other potential sources of infection should also be considered, given that Everett et al. [226] established a positive correlation between increased amounts of nitrogen used for apple fertilization and ABR incidence. Conversely, canopy density, as well as boron levels, was negatively correlated with disease occurrence. However, the impact of potassium was inconclusive, and no relationship was found between ABR and calcium. In contrast, Børve et al. [164] reported that the application of calcium through foliar sprays during summer reduced ABR occurrence in cold storage in Norway. In an earlier study conducted in the US, Biggs [227] examined the effectiveness of three calcium salts (calcium chloride, calcium propionate, and calcium silicate) in preventing ABR caused by *C. acutatum* and *C. gloeosporioides*. While calcium chloride inhibited the growth of *C. acutatum* germ tubes by 50% and *C. gloeosporioides* by 41% compared to the control, 80% and 48% effectiveness was noted for calcium propionate. In six field trials involving three weekly dilute applications of calcium solutions, lower infection incidence was noted in fruits treated with calcium salts before being inoculated with either *C. acutatum* or *C. gloeosporioides* conidia compared to control fruits. These experiments demonstrated that calcium salt application can be integrated into disease management programs [227].

Other recommended agrotechnical measures for apple orchard cultivation (both conventional and organic) include optimal planting density, row orientation in the direction of prevailing winds, the maintenance of grass–mulch systems between rows, timely irrigation, balanced fertilization, and regular pest, disease, and weed control [81,165,228].

### 13.3. Biological Control

Reliance on chemical fungicides during the growing season is no longer a common practice due to the emergence of resistance and adverse ecotoxicological effects. Moreover, in the European Union as well as in many other European countries, the use of fungicides after fruit harvest is prohibited, necessitating the application of biological control measures. Accordingly, research efforts are increasingly being directed toward the identification of organisms that are effective in the control of *Colletotrichum* spp. pathogens of apple, including antagonistic yeasts, fungi, actinomycetes, bacteria, and green marine algae, as well as plant extracts, essential oils, and enzyme inhibitors.

As a part of the study conducted by Boyd-Wilson et al. [229], the antagonistic effects of 44 isolates of different yeasts collected in New Zealand were tested against *C. acutatum* on harvested apple fruits. However, only four of these reduced the bitter rot lesions. Nonetheless, when yeasts were applied prior to inoculation, lesion size was significantly reduced compared to their application after the inoculation. The most effective reduction was achieved with living yeast cells that were washed and applied as a solution in sterile water without any nutritional supplements, whereas yeast extracts had no effect [229]. In an earlier study, Suzzi et al. [230] investigated the antagonistic effects of 12 strains of yeasts from the genus *Saccharomyces* and 4 strains from the genus *Zygosaccharomyces* isolated from grape berries when applied to 10 different pathogens, including *C. acutatum*. While all strains exhibited high efficacy against *C. acutatum*, a combination of these strains yielded the best biocontrol results, as each antagonistic yeast was characterized by distinct selective actions against the pathogen’s mycelium [230].

Antagonistic fungi that do not cause any symptoms on apple fruits have also been used as biocontrol agents. For example, Dharmaputra et al. [162] investigated the pathogenicity of various antagonistic fungi against *C. acutatum* and noted that *Aspergillus flavus* and *Fusarium graminearum*, two isolates from the genus *Pestalotiopsis* (*Pestalotiopsis* sp. 1 and *P. guepinii*), and three unidentified fungal isolates (R3, I3, and D3) caused 4–64% inhibition of *C. acutatum* growth. Only *F. graminearum* caused disease symptoms on the apple fruit, while *P. guepinii* application at a conidial suspension concentration of 4 × 10^6^ conidia/mL resulted in the greatest ABR inhibition (39.47%).

Lee et al. [231] tested the effectiveness of antagonistic bacteria in controlling *C. acutatum* infection in apples, and found that the S16 strain of *Bacillus subtilis* reduced the disease incidence by up to 80% under controlled conditions. However, data on the use of *B. amyloliquefaciens* in controlling diseases caused by *Colletotrichum* spp. on apples are presently limited.

The antagonistic effects of soil actinomycetes were tested by Sadeghian et al. [232] against the fungus *C. gloeosporioides* (the causative agent of ABR). Analyses conducted as a part of this study conducted in Iran indicated that six actinomycete isolates exhibited significant inhibitory properties on the mycelial growth of the pathogen, whereby *Amycolatopsis* sp. was the most effective isolate in in vitro biological tests.

Following their evaluation of the impact of ulvan—a water-soluble polysaccharide extracted from the green seaweed *Ulva fasciata* Delile—on managing GLS, Araújo and Stadnik [86] concluded that it was associated with the activity of the peroxidase and β-1,3-glucanase enzymes. The authors attributed the positive effect of ulvan (66% reduction in leaf disease incidence on both young and old leaves) to increased peroxidase enzyme activity. The enzyme β-1,3-glucanase exhibited similar effects on both resistant and susceptible seedlings.

In testing the effectiveness of various plant extracts in controlling ABR, Moline and Locke [233] noted the moderate efficacy of hydrophobic neem (*Azadirachta indica* A. Juss.) seed extract (clarified neem oil) against *Glomerella cingulata* when applied to Golden Delicious apple fruits (which were previously pressure-infiltrated with 2% CaCl_2_) in storage. These authors also observed an 80% reduction in ethylene production in apple fruits dipped in 2% neem seed oil compared to wounded, inoculated controls. However, neem seed oil did not produce statistically significantly better results when compared to 2% CaCl_2_ infiltration. According to the findings reported by Zivanov et al. [234], the root extract of the invasive plant *Asclepias syriaca* L. exhibited antimicrobial activity against *C. gloeosporioides*, while its antifungal activity against *C. acutatum*—a dominant apple pathogen in storage in Serbia—was only demonstrated under in vitro conditions.

Cinnamon and clove essential oils (EOs) have also shown significant potential as biocontrol agents for the prevention and control of *C. gloeosporioides*—the causative agent of ABR. Both in vitro and in vivo studies conducted by Wang et al. [235] in China demonstrated that fumigation with these EOs effectively limits fungal growth and reduces rot. Scanning electron microscopy (SEM) and transmission electron microscopy (TEM) observations further revealed that, as a result of EO application, the morphology of the mycelium and cellular ultrastructure was altered, suggesting that these EOs are capable of destroying the integrity and structure of cell membranes and major organelles. RNA sequencing and bioinformatics analyses showed that clove EO treatment compromises membrane integrity and biological function by affecting genes involved in the membrane components and transmembrane transport.

As a part of their work, Gregori et al. [236,237] focused on the capacity of enzyme inhibitors of protease (PT) and polygalacturonase (PG) produced by *C. acutatum*, extracted from stored apples, to mitigate *C. acutatum* infection. The authors also extracted protease inhibitors (PI) and polygalacturonase inhibitors (PGIP) from healthy stored apples. According to the in vitro radial diffusion assays, over 41% and 62% inhibition was achieved after 24 h for PI and PGIP, respectively. After four days at 20 °C, infection inhibition in inoculated fruit ranged from 33.9% to 54.4% for PI, and 23.5–45% was measured for PGIP after five days at 20 °C. More recently, Velho et al. [144] found significant differences in the extracellular enzyme production between *C. fructicola* isolates causing ABR and GLS. Although ABR isolates exhibited higher amylase and pectinase activity, there were no significant differences between isolates with respect to any of the tested enzymes during apple leaf infection. Thus, as no differences in extracellular enzymes between ABR and GLS isolates were observed, further research into these enzymes and their roles in the pathogenesis of *C. fructicola* on apples is needed.

To effectively control apple fruit decay in the post-harvest period, various alternatives to synthetic fungicides can be explored. For example, to control ABR caused by *C. acutatum* on Golden Delicious apples under controlled atmosphere conditions, Janisiewicz et al. [238] combined the antagonistic yeast *Metchnikovia pulcherrima* T5-A2 with heat treatments and 1-methylcyclopropene (1-MCP)—an ethylene receptor inhibitor that slows apple maturation. The authors noted that heat treatment had a limited effect on reducing ABR, while the isolate T5-A2 of *M. pulcherrima* proved effective in suppressing it even after the apples were subjected to heat treatment. On the other hand, 1-MCP treatment increased the ABR development. As the presence of the antagonist neutralized this effect, combining antagonist yeast treatment with heat treatment appears to be more effective in controlling ABR. The 1-MCP treatment also accelerated apple ripening, which increased ABR incidence, confirming the need for further research into the mechanisms by which 1-MCP affects rot development [238].

According to the Greenbook, two commercial biofungicides are recommended for the control of *Colletotrichum* spp.: (1) Serenade ASO containing *Bacillus subtilis* strain QST 713, commercialized by Bayer, and (2) Double Nickel 55 containing *Bacillus amyloliquefaciens* strain D747, which is commercialized by Certis [239]. Although numerous biological measures and biofungicides have been tested against *Colletotrichum* spp. on fruit crops, their field efficacy is presently unreliable [196]. Therefore, further field trials with these and other biological measures and commercial biofungicides are necessary to establish their effectiveness in controlling apple pathogens of the *Colletotrichum* genus.

### 13.4. Chemical Control

Cultural practices are the first step in the fight against ABR, but to keep the disease under control, they should typically be complemented by the use of fungicides, which have the potential to improve tree health and maximize apple yields [13]. Previously used fungicides—such as benomyl, captafol, chlorothalonil, mancozeb, thiophanate-methyl, and thiram—exhibit moderate to good efficacy against several plant diseases, including ABR caused by different species of the *Colletotrichum* genus [151,240,241]. However, owing to the increasingly stringent restrictions on the use of chemicals across the globe, focus has shifted to compounds that are less damaging to human and plant health, as well as the environment.

According to the spray guides for *Colletotrichum* control in the United States, seven chemical groups of fungicides with single-site modes of action should be adopted in the management of *Colletotrichum* spp. apple pathogens: (1) methyl benzimidazole carbamates (MBC) (FRAC 1); (2) demethylation inhibitors (DMI) (FRAC 3); (3) succinate dehydrogenase inhibitors (SDHI) (FRAC 7); (4) quinone-outside inhibitors (QoI) (FRAC 11); (5) phenylpyrroles (PP) (FRAC 12); (6) polyoxins (FRAC 19); and (7) fluazinam (FRAC 29) [196]. However, in a recent comparative study of fungicide efficacy, Martin et al. [242] established significant differences between and within FRAC groups, but noted that the frequency of resistant isolates in the Mid-Atlantic region of the United States was too low to affect the regional increase in ABR.

These findings are supported by the evidence provided by growers, who frequently report variability in fungicide efficacy against ABR, which Munir et al. [137] attributed to differences in fungicide sensitivity among *Colletotrichum* species and species complexes. Indeed, Dowling et al. [196] demonstrated that *Colletotrichum* spp. affecting fruit crops often vary in their sensitivity to fungicides such as FRAC 1, FRAC 3, and FRAC 11. These authors posited that such differences can be noted even within a single geographic location. While they may be inherent, they most likely arise as a result of fungicide selection pressure influenced by the abundance of species-specific inoculum, and the presence of wild-type phenotypes within nearby populations [196]. According to Yokosawa et al. [131], who investigated ABR in Japan, while none of the studied *C. siamense* isolates were resistant to MBC and QoI, *C. fructicola* isolates were often resistant to QoI only, or both MBC and QoI fungicides. *C. fioriniae* also exhibited higher sensitivity to difenoconazole, fludioxonil, and pyraclostrobin, and lower sensitivity to benzovindifupir and thiabendazole. Khodadadi et al. [5] also established that *C. noveboracense* isolates had higher EC50 values for difenoconazole and fludioxonil compared to *C. chrysophilum* isolates, which showed lower sensitivity to pyraclostrobin and benzovindifupir. Thus, understanding the fungicide resistance profiles of different ABR causative agents is essential for providing appropriate management recommendations to growers [196].

Sutton [13] indicated that preventive sprays containing dithiocarbamates, fluazinam, ditianon, captan, phosphite (Phi), and QoI fungicides are highly effective in ABR management. As 10 out of the 22 most commonly used fungicides and mixtures registered for managing *Colletotrichum* spp. on fruit crops are QoI or QoI mixtures, these findings are encouraging. On the other hand, overreliance on QoI has also contributed to fungicide resistance in many *Colletotrichum* spp. In the United States, *Colletotrichum* species resistant to FRAC 11 (QoI) fungicides have already been identified on several commercial fruit crops, including *Malus domestica* [196,243,244]. Munir et al. [137] are also of the view that, to prevent the development of resistance in *C. gloeosporioides* to MBC fungicides, they should not be the sole means of controlling this disease. These assertions are supported by Chechi et al.’s [244] report from Illinois, US, confirming the resistance of individual *C. siamense* isolates to both MBC and QoI. As DMI fungicides are widely used in fruit growing, either in isolation or in combination with other products (e.g., difenoconazole + cyprodinil), rotating these products with other chemical formulations, such as boscalid + pyraclostrobin or products from the SDHI fungicide group, should reduce the risk of resistance development while maintaining effective disease control [165,245].

However, to fully mitigate the risk of resistance, new fungicides need to be continually developed [246], but control strategies based on protective fungicides with multi-site modes of action, such as captan or ziram, have also been shown to be beneficial [243]. According to Abbot and Beckerman [247], the effectiveness of captan in controlling apple diseases (including ABR) can be further enhanced by adding certain adjuvants that increase water droplet coverage and thus improve the distribution of active ingredients on plants. However, as adjuvant performance depends on the environmental conditions as well as apple cultivar sensitivity, their selection is crucial for not only effective disease control but also reductions in phytotoxicity. Further research is thus needed to better understand the role of adjuvants in the performance of different fungicides under field conditions [247].

According to Trkulja [81,248], for successful apple fruit storage, it is particularly important to consider the timing and choice of final fungicide treatment before harvest.

### 13.5. Resistant Varieties

The management of agricultural plant diseases is most effectively achieved by the introduction of resistant varieties. However, the long breeding and selection period, along with the reliance on fungicides for quicker results, can compromise the utility of this approach.

Although no apple varieties are completely resistant to ABR, their tolerance varies considerably. For example, Shi et al. [249] noted significantly fewer and smaller lesions on the fruit of Granny Smith, Jonagold, Jonathan, Red Delicious, and Red Rome varieties compared to other tested apple genotypes, while Braeburn, Gala, and MacIntosh were the most susceptible to ABR pathogens. Biggs and Miller [250] classified apple varieties into four groups based on relative susceptibility: (1) Most Susceptible (Ginger, Honeycrisp, and Pristine); (2) Very Susceptible (Arlet, Enterprise, Sansa, and Yataka); (3) Moderately Susceptible (Creston, Golden Delicious, Golden Supreme, GoldRush, PioneerMac, and Sunrise); and (4) Least Susceptible (Fuji). According to Leonberger et al. [165], Arkansas Black, Cripps Pink/Pink Lady, Empire, Enterprise, Fuji, Gala, Ginger Gold, Golden Delicious, Honeycrisp, and Jonagold are particularly sensitive to ABR pathogens. On the other hand, Jílková and Víchová [251] found that Braeburn, Jonagold, and Rubinola were among the apple varieties with a lower susceptibility to tested isolates of the *C. acutatum* species complex, while Jonagored and Otava were among the most susceptible. In the United States, Honeycrisp and Empire are recognized as some of the most susceptible varieties to ABR [4,139]. Khodadadi et al. [6] concurred with this finding, noting that *M. sylvestris* (accession PI 369855) was the most resistant among the tested apple varieties. While ABR on Gala and Golden Delicious are frequently reported [4,68,139,146], according to Onofre and Antoniazzi [176], in Brazil, Gala showed the highest resistance, while Golden Delicious was the most susceptible to ABR. Bitter rot was also noted on Fuji in New York State, US [4], but also in China [127]. Crimson Crisp, Enterprise, Granny Smith, Idared, Jonathan, and McIntosh were also among the varieties affected by ABR in the United States [4,139,151], while in Argentina, this disease has been identified on Eva, Carica, and Princese [150]. In Belgium, ABR has been reported on Pinova and Nicoter, and in the Netherlands, on Aroma [98,164].

Based on the study conducted in Bosnia and Herzegovina aiming to assess the susceptibility of 35 apple varieties or rootstock variants to selected isolates of *C. acutatum*, Trkulja [252] noted that all tested varieties were susceptible to the selected *C. acutatum* isolates. However, there were statistically significant differences in susceptibility between different apple varieties, whereby Prima showed the highest susceptibility, while the local variety Paradija (also known as Dugostajka in the Potkozarje area where it is grown) exhibited the highest resistance. The author further established that the rootstock on which the varieties were grown played a substantial role in the susceptibility to the studied *C. acutatum* isolates.

In an earlier study, Camilo et al. [178] similarly observed high levels of resistance to *Glomerella cingulata* in several wild apple species, including *Malus zumi* var. *calocarpa*, *M. prunifolia* var. *xanthocarpa* NA 3604, *M. x sieboldii* 301, *M. purpurea* ‘Lemoine’, *M. x sieboldii* AA 852, *M. nieuwlandiana*, NY-78231-3, and NY-78231-6. While none of the tested species exhibited immunity, large-fruited clones such as NY-53710-95, NY-55, and Red Rome also demonstrated high levels of resistance to ABR [178]. According to Nita et al. [205], incubation temperature also plays a role in the ABR susceptibility of different apple varieties. For example, Idared may be more susceptible to *C. siamense* under warmer conditions, whereas Golden Delicious becomes more resistant.

Apple varieties also differ in their susceptibility to *Glomerella* leaf spot. First reported on Granny Smith [128], it was later identified on the leaves of Eva [76]. Since then, GLS has been confirmed on Golden Delicious leaves in the US [65], but epidemics of this disease have also occurred in Gala orchards [67]. An ample body of evidence indicates that the varieties from the Golden group (Cripps Pink, Cripps Red, Gala, Galaxy, and Golden Delicious) are highly susceptible to GLS, whereas those from the Red Delicious group (such as Fuji), exhibit complete or partial resistance [71,86,201,253]. In Uruguay, where varieties from the Red Delicious and Spur groups are most commonly grown, GLS used to be absent [71]. However, its subsequent detection suggests that these varieties are not fully immune to GLS [88,188]. Indeed, pathogenicity tests conducted in China by Wang et al. [73] showed that species from the genus *Colletotrichum* can infect the leaves of Golden Delicious, as well as Gala, Golden Centuri, Honeycrisp, Kinguan, Pacific Rose, and Pink Lady, but their pathogenicity depends on the apple variety. Severe foliar disease in China was also reported by Chen et al. [7], where not only Golden Delicious and Gala, but also Jonagold, was affected.

### 13.6. Apple Fruit Protection in Storage

As the ABR resistance of apple fruits in storage is associated with several factors—including the variety, ripening period, fruit firmness, chemical composition of the fruit (such as acidity, sugars, and polyphenols), and changes in chemical composition caused by infection—different apple varieties exhibit resistance or susceptibility to various ABR-causing fungi [254]. According to Shi et al. [255], fruit sensitivity also increases with age, whereas Ahmadi-Afzadi et al. [256] emphasized the role of harvest timing, as greater fruit firmness at harvest and less pronounced softening processes during storage reduce disease susceptibility.

Thus, various measures need to be taken for the successful preservation of stored apple fruits, including optimal harvest timing and method, as well as subsequent handling, sorting, and packaging. According to Trkulja [78,81,228], the timing of harvest is crucial, as the maturity level significantly influences fruit storage success. For example, fruits intended for cold storage should be harvested slightly before full ripeness, as they are less prone to mechanical damage and pathogen attacks and are easier to transport at this stage. Additionally, various injuries that inevitably occur during harvesting—such as punctures, bruises, and abrasions—should be minimized, and damaged fruit should be removed prior to storage if possible. Strict adherence to hygiene measures during sorting and packaging is also essential to avoid inoculum spreading to healthy fruit during and after storage. Most importantly, diseased apples should be removed during sorting to prevent them from serving as a new source of inoculum.

The primary objective of modern storage technology is complete control over the respiration, ripening, and aging processes of apple fruits, while maintaining their vitality. This strategy increases their natural resistance to *Colletotrichum* spp. and enhances their ability to heal wounds. Accordingly, controlled atmosphere (CA) storage rooms, where CO_2_ concertation is elevated while oxygen levels are extremely low, are increasingly utilized for preserving harvested apples. However, it is also important to determine and adopt the optimal long-term storage parameters for each apple variety [78,81]. Contemporary apple storage methods rely on a combination of post-harvest heating and CA storage as a means of significantly reducing the disease incidence without any adverse impacts on apple quality. Tahir et al. [257] have found that this approach can reduce ABR occurrence by 65% and 73% for the Ingrid Marie and Aroma varieties, respectively, compared to untreated apples.

## 14. Conclusions

This review provides a comprehensive and up-to-date profile of ABR and GLS, diseases caused in apple fruit and leaves, respectively, by fungi from the genus *Colletotrichum*. Currently, available molecular tools have enabled the discovery of 27 distinct *Colletotrichum* species responsible for ABR, as well as 15 causative agents of GLS. These species generally belong to one of three species complexes—*C. acutatum*, *C. gloeosporioides*, and *C. boninense*—and are differentiated by geographical distribution, capacity to cause ABR and/or GLS, pathogenicity with respect to different apple varieties, epidemiological characteristics, sensitivity to different fungicides, and frequency of resistant isolate emergence, among other factors. However, data on the interactions between individual apple varieties and the members of *Colletotrichum* species complexes that produce ABR and GLS are still limited. Differences in fungicide susceptibility among *Colletotrichum* species from one geographic location may be inherent or may arise as a result of fungicide selection pressures influenced by species-specific inoculum frequency and density, as well as the influx of wild-type phenotypes from nearby hosts. In addition, significant differences in the sensitivity of individual apple varieties to different species of the genus *Colletotrichum* to the causative agents of ABR and GLS were determined. Likewise, some species of the genus *Colletotrichum* react differently to the applied control measures. Consequently, the correct identification of *Colletotrichum* species that cause ABR and GLS is very important for the development of effective control management and profitable apple cultivation.

## Figures and Tables

**Table 1:** List of genes, primer pairs, their sequences, amplicon length and references for the identification of *Colletotrichum* species.

Gene	Primer Name	Sequence	Amplicon Length (bp)	Reference
ITS	ITS1	TCCGTAGGTGAACCTGCGG		
	ITS4	TCCTCCGCTTATTGATATGC	610	[210]
	ITS5	GGAAGTAAAAGTCGTAACAAGG	-	
	CaInt2	GGGGAAGCCTCTCGCGG	490	[211]
	CgInt	GGCCTCCCGCCTCCGGGCGG	450	
TUB2	T1	AACATGCGTGAGATTGTAAGT	1500	[213]
	T2	TAG TGA CCC TTG GCC CAGT TG		
	Bt2b	ACCCTCAGTGTAGTGACCCTTGGC	500	[212]
	Bt2a	GGTAACCAAATCGGTGCTGCTTTC		
GAPDH	GDF1	GCCGTCAACGACCCCTTCATTGA	270	[172]
	GDR1	GGGTGGAGTCGTACTTGAGCATGT		
TUB	TB5	GGTAACCAGATTGGTGCTGCCTT	550	[219]
	TB6	GCAGTCGCAGCCCTCAGCCT		
ACT	ACT-512F	ATGTGCAAGGCCGGTTTCGC	270	[216]
	ACT-783R	TACGAGTCCTTCTGGCCCAT		
CHS-1	CHS-345R	TGGAAGAACCATCTGTGAGAGTTG	300	[216]
	CHS-79F	TGGGGCAAGGATGCTTGGAAGAAG		
HIS3	CYLH3F	AGGTCCACTGGTGGCAAG	-	[217]
	CYLH3R	AGCTGGATGTCCTTGGACTG	-	
TUB	BT2Fd	GTBCACCTYCARACCGGYCARTG	333	[214]
	BT4R	CCRGAYTGRCCRAARACRAAG		
CAL	CL1C	GAATTCAAGGAGGCCTTCTC	830	[10]
	CL2C	CTTCTGCATCATGAGGTGGAC		
GS	GSF	ATGGCCGAGTACATCTGG	900	[218]
	GSR	GAACCGTCGAAGTTCCAC		
ApMat	CgDL-F6	AGTGGAGGTGCGGGACGTT	870	[185]
	CgMAT1F2	TGATGTATCCCGACTACCG		
APN2	ColDL-F3	GGGAGAAGCGAACATACCA	900	[185]
	CgDL-R1	GCCCGACGAGCAGAGGACGTAGTC		

## Data Availability

Not applicable.
